# Recurrent co-domestication of *PIF/Harbinger* transposable element proteins in insects

**DOI:** 10.1186/s13100-022-00282-2

**Published:** 2022-11-30

**Authors:** Dragomira N. Markova, Fatema B. Ruma, Claudio Casola, Ayda Mirsalehi, Esther Betrán

**Affiliations:** 1 Department of Biology, University of Texas at Arlington, Arlington, TX USA; 2 Department of Ecology and Conservation Biology, Texas A&M University, College Station, TX USA

**Keywords:** PIF/harbinger transposable element, Molecular domestication, Insects

## Abstract

**Background:**

Transposable elements (TEs) are selfish DNA sequences capable of moving and amplifying at the expense of host cells. Despite this, an increasing number of studies have revealed that TE proteins are important contributors to the emergence of novel host proteins through molecular domestication. We previously described seven transposase-derived domesticated genes from the *PIF/Harbinger* DNA family of TEs in *Drosophila* and a co-domestication. All *PIF* TEs known in plants and animals distinguish themselves from other DNA transposons by the presence of two genes. We hypothesize that there should often be co-domestications of the two genes from the same TE because the transposase (gene 1) has been described to be translocated to the nucleus by the MADF protein (gene 2). To provide support for this model of new gene origination, we investigated available insect species genomes for additional evidence of *PIF* TE domestication events and explored the co-domestication of the MADF protein from the same TE insertion.

**Results:**

After the extensive insect species genomes exploration of hits to *PIF* transposases and analyses of their context and evolution, we present evidence of at least six independent *PIF* transposable elements proteins domestication events in insects: two co-domestications of both transposase and MADF proteins in *Anopheles* (Diptera), one transposase-only domestication event and one co-domestication in butterflies and moths (Lepidoptera), and two transposases-only domestication events in cockroaches (Blattodea). The predicted nuclear localization signals for many of those proteins and dicistronic transcription in some instances support the functional associations of co-domesticated transposase and MADF proteins.

**Conclusions:**

Our results add to a co-domestication that we previously described in fruit fly genomes and support that new gene origination through domestication of a *PIF* transposase is frequently accompanied by the co-domestication of a cognate MADF protein in insects, potentially for regulatory functions. We propose a detailed model that predicts that *PIF* TE protein co-domestication should often occur from the same *PIF* TE insertion.

**Supplementary Information:**

The online version contains supplementary material available at 10.1186/s13100-022-00282-2.

## Background

Transposable elements (TEs) are selfish genetic elements found in nearly all eukaryotes that are able to move and replicate within the genome. In some groups of organisms, like mammals and grasses, TEs represent the single largest component of the genome, accounting for 40 to 85% of the nuclear DNA respectively [1–4]. Eukaryotic TEs are usually divided into two main classes according to their mechanism of transposition. Class I or retroelements move via an RNA intermediate that is reverse-transcribed, while class II elements or DNA transposons move directly as DNA. In a typical DNA transposition reaction and after transcription and translation, the transposase relocates into the nucleus and binds in a sequence-specific manner to the terminal inverted-repeats (TIRs) located at each end of the transposon and catalyzes both the DNA cleavage and strand transfer steps of the reaction [5, 6].

The *P Instability Factor* (*PIF*) superfamily of DNA transposons, also known as *Harbinger* superfamily [7] is widespread in plants and animals. Contrary to most other superfamilies of DNA transposons, *PIF/Harbinger* transposable elements (hereafter, *PIF* elements) are characterized by the presence of two protein-coding genes [8–13]. One of these genes encodes a ~ 400–500-aa protein representing the catalytic transposase, while the other gene encodes a protein of ~ 300–400 aa with a Myb-like domain (also known as SANT/trihelix or MADF domain [10, 13]). Although the transposition mechanism of *PIF* transposons has not been thoroughly characterized, molecular and genetic studies of rice [14, 15] and zebrafish [16] *PIF* elements indicate that both proteins are required for transposition. For zebrafish *Harbinger3_DR*, it is also known that the Myb-like protein interacts physically with the transposase and this interaction is necessary for the colocalization of both proteins to the nucleus [16]. Both the Myb-like and the transposase proteins of *PIF* elements are predicted to contain DNA-binding domains (Myb/SANT/MADF and HTH, respectively [9–11, 13]); however this activity has only been experimentally demonstrated for the Myb-like protein encoded by *Harbinger3_DR*, which recognizes specifically a 9-bp motif located in the subterminal regions of the transposon [16].

One of the most direct contributions of TEs to their host genomes occurs with the process of ‘molecular domestication’ whereby the gene (or genes) encoded by and serving the replication of a TE is (are) co-opted by the host genome to create a new gene (new genes) with cellular function(s) [6, 17–22]. Recent studies suggest that TE domestication is a common pathway for the emergence of new genes and functions [6, 19–24]. For example, there are over a hundred human genes entirely or partially derived from TE coding sequences [1, 6, 25, 26].

In *PIF* TEs, both the transposase and the Myb-like protein have been co-domesticated multiple times from independent invasions in eukaryotic genomes. In vertebrates, the *PIF*-like superfamily of transposases has given rise to a domesticated gene known as *HARBI1*, which is highly conserved from teleosts to mammals [11, 13], but has yet to be functionally characterized. Sinzelle et al. showed that HARBI1 interacts physically with the Myb-like protein NAIF1 (nuclear apoptosis-inducing factor 1) and that this interaction is required for the nuclear localization of HARBI1 in human cells [16]. The hypothesis is that both proteins were co-domesticated from the same *PIF* transposon family to serve in the same, yet to be identified, cellular pathway [16]. Although overexpression of *NAIF1* is known to inhibit cell growth and induce apoptosis [27], its physiological function still remains unknown.

In *Arabidopsis,* several cases of potential co-domestication of *PIF* TE proteins have also been published. The domesticated *PIF*-like transposase ALP1 interacts with the Myb domain-containing protein ALP2 to modulate the activity of the Polycomb Repressor Complex 2 (PRC2), which in *Arabidopsis* is known to contribute to epigenetic transposon silencing [28, 29]. *ALP1* and *ALP2* were shown both to have an ancient origin in land plants and likely derive from the co-domestication of a single *PIF*-element [28, 29].

A second *Arabidopsis PIF* TE domesticated transposase, HDP1, has been shown to physically interact with the Myb-like protein HDP2. HDP1 and HDP2 are associated with a histone acetyltransferase complex leading to DNA demethylation and have a role in chromatin opening [30].

In *Drosophila,* we have previously reported one likely case of co-domestication wherein the *PIF*-like transposase gene *DPLG7* and the Myb-like gene *DPM7* reside next to each other in the genome, following a single transposon insertion [13].

Given that Myb-like proteins can localize in the nucleus and bind to DNA in the absence of the transposase [15, 16], these cases of co-domestication suggest that the presence of cognate Myb-like proteins might be required in order for *PIF* transposases to be recruited as host genes [13] and we expect a correlation between the presence of domesticated *PIF* transposases and MADF proteins in the genomes.

To understand how often new genes originate from *PIF* TEs and test our co-domestication hypothesis, we investigated available insect genomes for additional cases of *PIF* TE domestication events. We present evidence of at least six *PIF* TE proteins domestication events in insects: two co-domestications of both transposase and MADF proteins in *Anopheles* (Diptera), one transposase-only domestication event and one co-domestication in butterflies and moths (Lepidoptera), and two transposase-only domestication events in cockroaches (Blattodea). Our results show that domestication of *PIF* transposases is frequently accompanied by the co-domestication of a cognate MADF protein from the same *PIF* TE insertion, potentially for regulatory functions. There are several features, including dicistronic transcription and complementary predicted nuclear localization signal between the two *PIF* TE genes, that support a model of how the co-domestications take place and reveal that *PIF* TEs are a recurrent source of multiple new host genes being domesticated at the same time.

## Results and discussion

### Domestication and co-domestication events of both *PIF* transposase and MADF protein in insect genomes

To retrieve sequences related to *PIF* transposases, we performed reiterative tBLASTn and BLASTp searches of insect NCBI proteins and translated nucleotide databases using the seven previously identified *PIF* domesticated transposases from *Drosophila*, i.e., DPLG proteins, as initial queries and all hits to *PIF* TE related sequences collected in the searched insect genomes. This approach added up to more than 250 hits to *PIF* transposases being used as queries (see Methods).

**Fig. 1:**
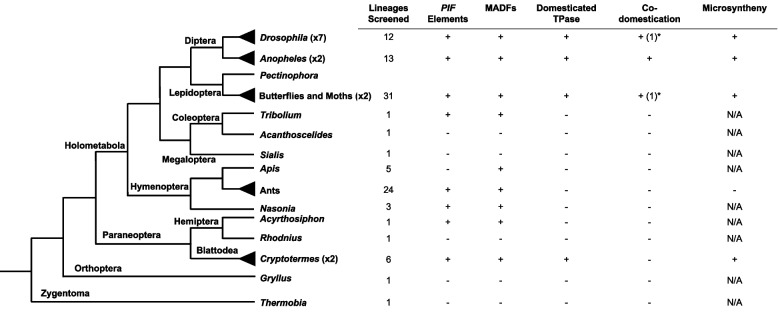
Phylogenetic relationship between representative insect genera and number of lineages/genomes screened within each group. Asterisks are shown in taxa where both transposase gene-only domestications and co-domestications of both genes occur, with the latter indicated in brackets

We screened all species with sequenced and annotated genomes in NCBI within (in alphabetic order): Archaeognatha, Blattodea, Coleoptera, Collembola, Dermaptera, Diplura, Diptera, Ephemeroptera, Hymenoptera, Lepidoptera, Megaloptera, Odonata, Orthoptera, Phasmatodea, Plecoptera, Psocodea, Siphonaptera, and Thysanoptera. We couldn’t screen (in alphabetic order): Embioptera, Grylloblattodea, Mantodea, Mantophasmatodea, Mecoptera, Neuroptera, Protura, Raphidioptera, Zoraptera and Zygentoma (Thermobia) because of lack of sequenced, annotated genomes. See Methods for more details and summary Fig. 1 and Supplementary Table 1.

We confirmed the domestications previously found in *Drosophila* (*DPLG1–7*) and dated their time of domestication with higher accuracy (See Methods). We obtained good support for domestications events within *Anopheles* (Diptera), Lepidoptera and Blattodea. We present all the evidence below of six additional *PIF* TE proteins domestication events in insects, in separate sections. First, two co-domestications of both transposase and MADF proteins in *Anopheles* (Diptera), then one transposase-only domestication event and one co-domestication in butterflies and moths (Lepidoptera), and lastly two transposase-only domestication events in cockroaches (Blattodea). Our inferences are based on the genes being in syntenic regions in the genomes in far related species, under purifying selection and having lost the hallmarks of being active TEs, i.e., absence of related copies in the genomes and TIRs, following our previous work approach [13]. See details below and in Methods.

### Two co-domestication events of both *PIF* transposase and MADF protein in *Anopheles*

**Fig. 2:**
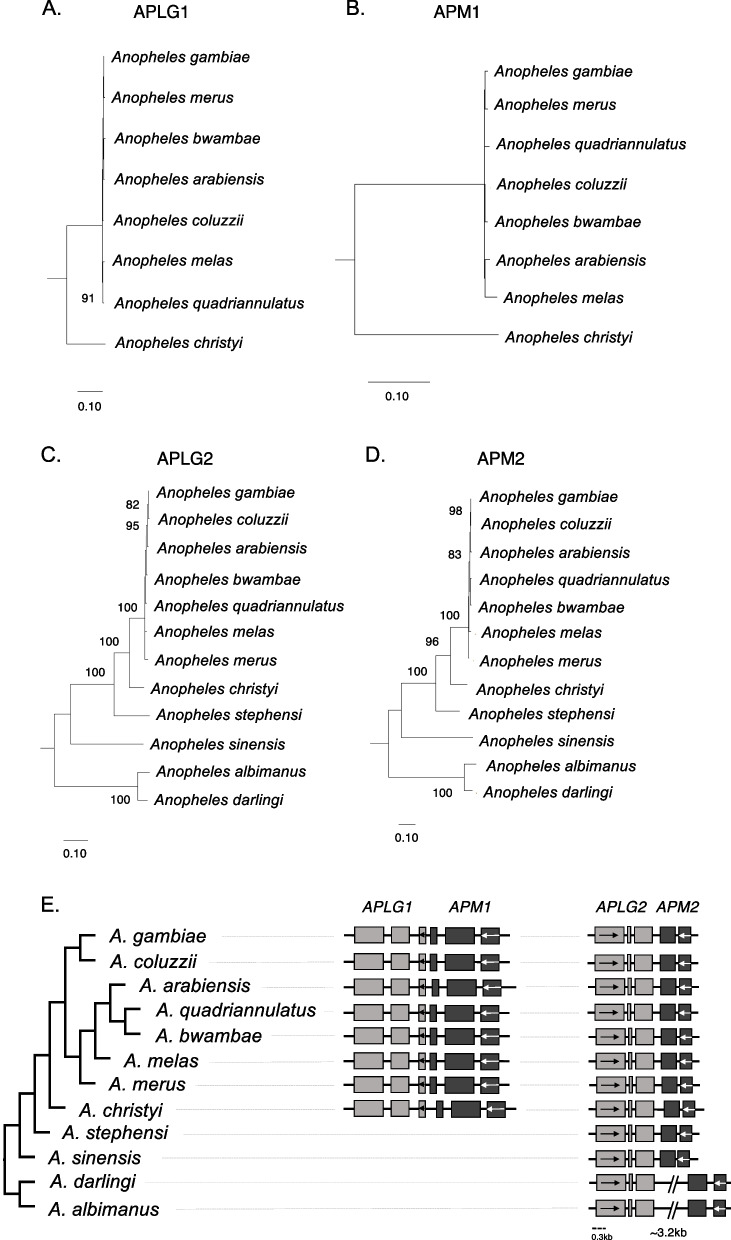
Maximum likelihood phylogeny and structure of *Anopheles PIF*-like genes. **A** and **B**. Phylogenetic relationship of *Anopheles PIF*-like domesticated proteins, APLG1 and APM1, respectively. **C** and **D**. Phylogenetic relationship of *Anopheles PIF*-like domesticated proteins APLG2 and APM2, respectively. Bootstrap values equal to or above 80 are shown. (**E**). The genomic structure of the genes encoding for the transposase and the MADF protein are represented below the phylogenies. Shaded rectangles indicate exons, with arrows pointing to the direction of transcription. Introns and intergenic regions are shown as black lines. The two pairs of co-domesticated genes are next to each other but not always transcribed in the same direction. *APLGs* and *APMs* are in close physical proximity in most species but not all

The searches and the exploration of the genomic regions containing *PIF* transposases BLAST hits in *Anopheles* resulted in the identification of two unrelated potentially domesticated transposases (Fig. 2 and Supplementary Table 1). Upon further examination both cases appear to be analogous to the *PIF* co-domestication previously observed [13], i.e., *DPLG7* and *DPM7*, where both open reading frames of the same *PIF* TE insertion in *Drosophila* were domesticated. We named the first gene of the two *Anopheles* co-domestications as *Anopheles PIF-Like Gene 1* (*APLG1*) and *APLG2*, respectively, and the second gene as *Anopheles PIF MADF-like protein-encoding gene 1* (*APM1*) and *APM2* (Fig. 2). *APLG1* and *APM1* are 97 bp apart,

whereas the distance between *APLG2* and *APM2* is 179 bp in the *Anopheles gambiae* genome and the structures are well conserved in other species although the genes are much further apart in some lineages (Fig. 2E).

Multiple pieces of evidence support these examples as independent co-domestications in mosquito. We found that in both cases the transposase and the MADF proteins possess intact coding regions in all species of *Anopheles* we found hits in (Supplementary Table 1 and Supplementary file 1). They all appear as a single-copy gene in all species examined, supporting the hypothesis that they represent conserved orthologs. Additionally, *APMs* are in a region immediately adjacent to *APLGs* in all species analyzed (Fig. 2), suggesting that both proteins are derived from the domestication of the genes of the same transposon insertion. However, we did not find TIRs or target site duplications (TSDs) further supporting that these are not *PIF* TEs (see Methods). After manually annotating all retrieved sequences, we performed DNA multiple alignments and found that the exon-intron structure of each gene is also well conserved in all *Anopheles* species where we find the genes. Analyses of approximately 10 kb upstream and downstream of each genes revealed that the microsynteny of each region respectively is well-conserved (Supplementary Fig. 1). To examine the evolutionary relationship of both transposases and their respective MADF proteins, we performed phylogenetic analyses using the maximum likelihood (ML) approach (Fig. 2A-D), and found that in both cases the evolutionary history of domesticated proteins follows the evolutionary history of the *Anopheles* genus (Fig. 2 [31–33], providing further support that those proteins have been domesticated and are an integral part of the functionality of their host genomes. In addition, we estimated dN/dS ratios of all genes and maximum likelihood ratio tests indicated that purifying selection is the major evolutionary force acting on the transposase genes, i.e., dN/dS is statistically significantly smaller than 1 (*APLG1* dN/dS = 0.282; *APLG2* dN/dS = 0.330; LRT *P*-value < 0.05 for both genes), while MADF sequences can evolve sometimes much faster albeit under purifying selection with dN/dS ratios significantly smaller than 1 (*APM1* dN/dS = 0.517; *APM2* dN/dS *=* 0.074; LRT *P*-value < 0.05 for both genes; See Supplementary Table 2 and Fig. 1) consistent with the mode of evolution of these genes described in other species [28, 29]. dN/dS values are similar between *APLG1* and *APLG2*, suggesting that different *PIF* transposases might experience similar degrees of selective constraint in the same species.

Interestingly, *APLG1* and *APM1* in *A. gambiae* are annotated as a single transcript and the five annotated introns have good RNA-Seq support (Supplementary Fig. 2). *APLG1* and *APM1* tissue expression supports their functionality and co-expression in multiple tissues (Supplementary Table 3). *APLG2* and *APM2* transcription was also confirmed in *A. gambiae* for multiple tissues (Supplementary Table 3). These data support the functionality of these domesticated *PIF* genes. The dicistronic transcription of *APLG1* and *APM1* provides additional/potential justification for why co-domestication of transposase and MADF protein from the same insertion occurred.

Based on the previously established phylogeny of *Anopheles* [31] and the presence-absence of the domestications in different genomes (Fig. 2), we estimated that *APLG1* and *APM1* co-domestication is a relatively young co-domestication that occurred ~ 28 Mya. On the other hand, *APLG2* and *APM2* co-domestication is present in all *Anopheles* species supporting that these domestication took place approximately 100 Mya (http://www.timetree.org).

In the closely related species *A. darlingi*, *A. albimanus* and *A. aquasalis*, a further pair of genes encoding for a transposase and for a protein containing a MADF domain was found adjacent to *APLG2*. Given the genomic location, it is likely that these two genes derive from a tandem duplication of the pair *APLG2-APM2*; therefore, we named them *APLG2b* and *APM2b*. However, both pairs *APLG2-APLG2b* and *APM2-APM2b* show only 57% of protein sequence identity, suggesting that *APLG2b* and *APM2b* may have evolved rapidly after arising via duplication. The alternative scenario of an independent domestication event originating *APLG2b* and *APM2b* seems unplausible because of the aforementioned proximity of the two pairs of genes.

### Domestication and co-domestications of *PIF* TE genes in Lepidoptera

**Fig. 3:**
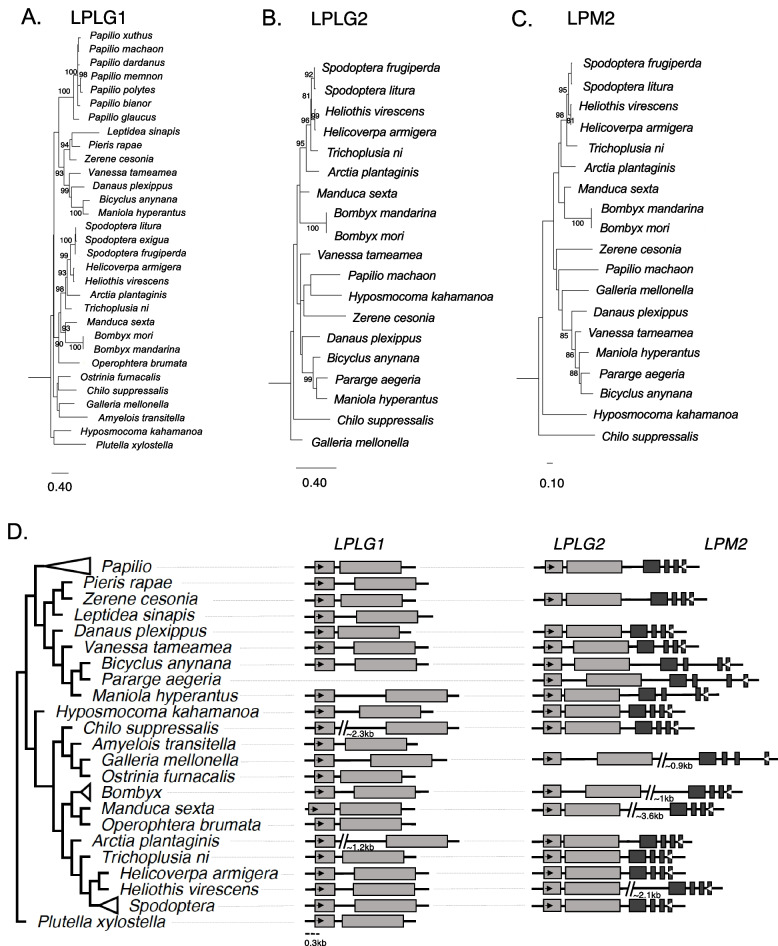
***A****. maximum* likelihood phylogeny and structure of the domesticated transposase gene *LPLG1* detected in 31 lepidopteran genomes. **B**. Maximum likelihood phylogeny of the transposase-encoding gene *LPLG2* from 18 lepidopteran genera. **C**. Phylogenetic relationships of the MADF-encoding gene *LPM2* in 18 lepidopteran genera. Bootstrap values equal to or above 80 are shown. **D**. Gene structures are shown for major groups. Exons in gene structures are shown as shaded rectangles. *LPLG2* and *LPM2* are in close physical proximity in most species but not all

We found two additional cases of *PIF* transposase domestications in several lineages of Lepidoptera (butterflies and moths). First, we identified a transposase domestication event in 31 Lepidoptera species, representing 21 genera, whose genomes are sequenced and annotated and publicly available, which we named *LPLG1* (*Lepidoptera PIF-like Gene 1*). Second, we found an additional case of an independently domesticated transposase, named hereafter *LPLG2* (*Lepidoptera PIF-like Gene 2*), in species representing 18 Lepidoptera genera (only data for representatives of the different genera is provided for this case). Both cases of domestications seem to have occurred at least 140 Mya (Akito et al. 2019; Figs. 1 and 3). BLASTp searches showed highly conserved orthologous sequences for each gene across genera (*E*-value = 0) and the lack of the structural hallmarks of active TEs (TIRs and TSDs). Both *LPLG1* and *LPLG2* are present as different single-copy genes within each genome examined, confirming the independent domestication origin of those transposases from a different *PIF* TE family. *LPLG1* and *LPLG2* both possess intact coding regions, composed of 385–428 aa and 356–402 aa, respectively (Supplementary file 1) and are under strong purifying selection (*LPLG1* dN/dS = 0.037; *LPLG2* dN/dS = 0.038; LRT *P* values < 0.05; Supplementary Table 2). Detailed examination of the genomic regions upstream and downstream of *LPLG1* and *LPLG2* showed a high degree of conservation across species and genera (Supplementary Fig. 1). Immediately adjacent to *LPLG2*, we found a gene encoding a MADF protein, named hereafter *LPM2* (*Lepidoptera MADF-like Gene 2*; Fig. 3 and Supplementary file 1), indicating a likely co-domestication of both *PIF* genes from the same *PIF* TE insertion in this case. *LPM2* orthologs have evolved under purifying selection (dN/dS = 0.1758; LRT *P*-value < 0.05 (Supplementary Table 2). *LPM2* is distant from *LPLG2* in a few lineages (Fig. 3D). However, we did not find TIRs or target site duplications (TSDs) further supporting that these are not *PIF* transposons (see Methods). To observe the evolutionary relationship of both transposases and the MADF protein, we performed ML phylogenetic analyses (Fig. 3A-C) and observe that the relationships follow quite closely the known phylogeny (Fig. 3 and [34, 35] but not completely. That gene trees do not always follow completely the species phylogeny is expected from incomplete lineage sorting [36, 37].

Transcription data in *Bombyx mori* reveals transcription of *LPLG2* and *LPM2* in all adult tissues analyzed, as well as expression of *LPLG1* in most adult tissues analyzed. While *LPLG2* and *LPM2* are transcribed in the same tissues, *LPLG2* shows lower levels compared to *LPM2* (Supplementary Table 3). Transcription of these genes support their functionality and in the co-domestication where we observe transcription in the same tissues of both genes albeit at different levels provides supports for at least partial coregulation of the two genes likely derived from the co-domestication of transposase and MADF protein from the same *PIF* TE insertion.

### Domestication of *PIF* TE genes in cockroaches

**Fig. 4:**
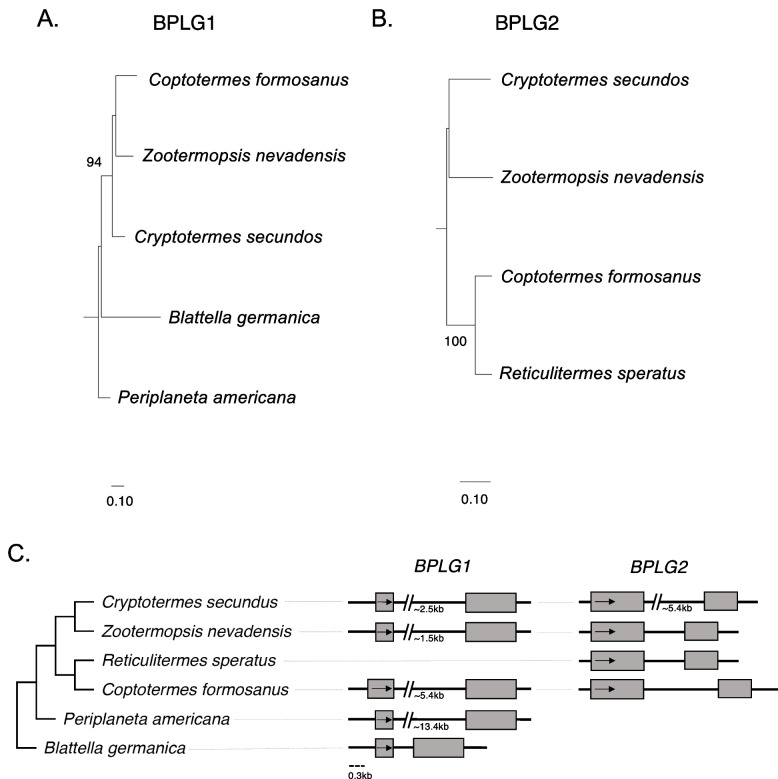
Maximum likelihood phylogeny and structure of Blattodea *PIF*-like genes. **A**. Phylogeny and structure of the domesticated transposase gene *BPLG1* present in five Blattodea species. **B**. Phylogeny and structure of the domesticated transposase gene *BPLG2* present in four Blattodea species. Bootstrap values equal to or above 80 are shown. **C**. Gene structures are shown. Shaded rectangles indicate exons, with arrows pointing to the direction of transcription. Introns are shown as black lines

In the superorder Paraneoptera, order Blattodea (cockroaches), we discovered two cases of *PIF* transposase-only domestication events. We named these genes *BPLG1* and *BPLG2*, for *Blattodea PIF-like Gene 1* and *2*. *BPLG1* and *BPLG2* were found in five and four species, respectively (Supplementary Table 1). *BPLG1* shows an intact coding region encoding a transposase-like protein of 378–401 aa (Supplementary Table 1 and Supplementary file 1) and maintains a conserved two-exons structure across all species although the single intron has change quite a bit in length (Fig. 4C). The regions flaking *BPLG1* show no evidence of the TIRs or TSD associated to *PIF* TE insertions. Analysis of the coding region suggests that this transposase-derived gene has been evolving under strong purifying selection (*BPLG1* dN/dS = 0.095; LRT *P*-value = 0, see Supplementary Table 2). Furthermore, examination of the genomic regions upstream and downstream of *BPLG1* shows a high degree of conservation across species (Supplementary Fig. 1). The gene phylogenies (Fig. 4A-B) are consistent with the known species phylogeny [38–40]. Overall, these findings support the scenario of a domestication event from a *PIF* transposase that is conserved across several species of cockroaches. Given its distribution we estimate that this domestication event occurred ~ 228 Mya (http://www.timetree.org), the oldest event among the five cases we identified.

*BPLG2* orthologs encode for a highly conserved 394–395 aa long transposase-like protein sharing 77–90% sequence identity. The gene contains two exons separated by an intron spanning ~ 900–1500 bp in all species except for the ~ 5400 long intron in *Cryptotermes secundus* (Fig. 4). No TIRs or TSD flanking *BPLG2* were identified and microsynteny data validated the orthology across species (Supplementary Fig. 1). *BPLG2* is under purifying selection (*BPLG2* dN/dS = 0.2486; LRT *P*-value < 0.001, see Supplementary Table 2). Given the species distribution the time of this domestication was 132 Mya (http://www.timetree.org). Although we did not find available genome wide expression data for these species, the two domestications in cockroaches are well supported at the sequence level.

Interestingly, multiple *PIF* TEs and MADF proteins are present in the annotated cockroach genomes (Fig. 1), but we did not observe the domestication of genes encoding MADF proteins next to the domesticated transposases.

### Evolutionary relationships of transposase sequences from *PIF*-like genes and transposons

**Fig. 5:**
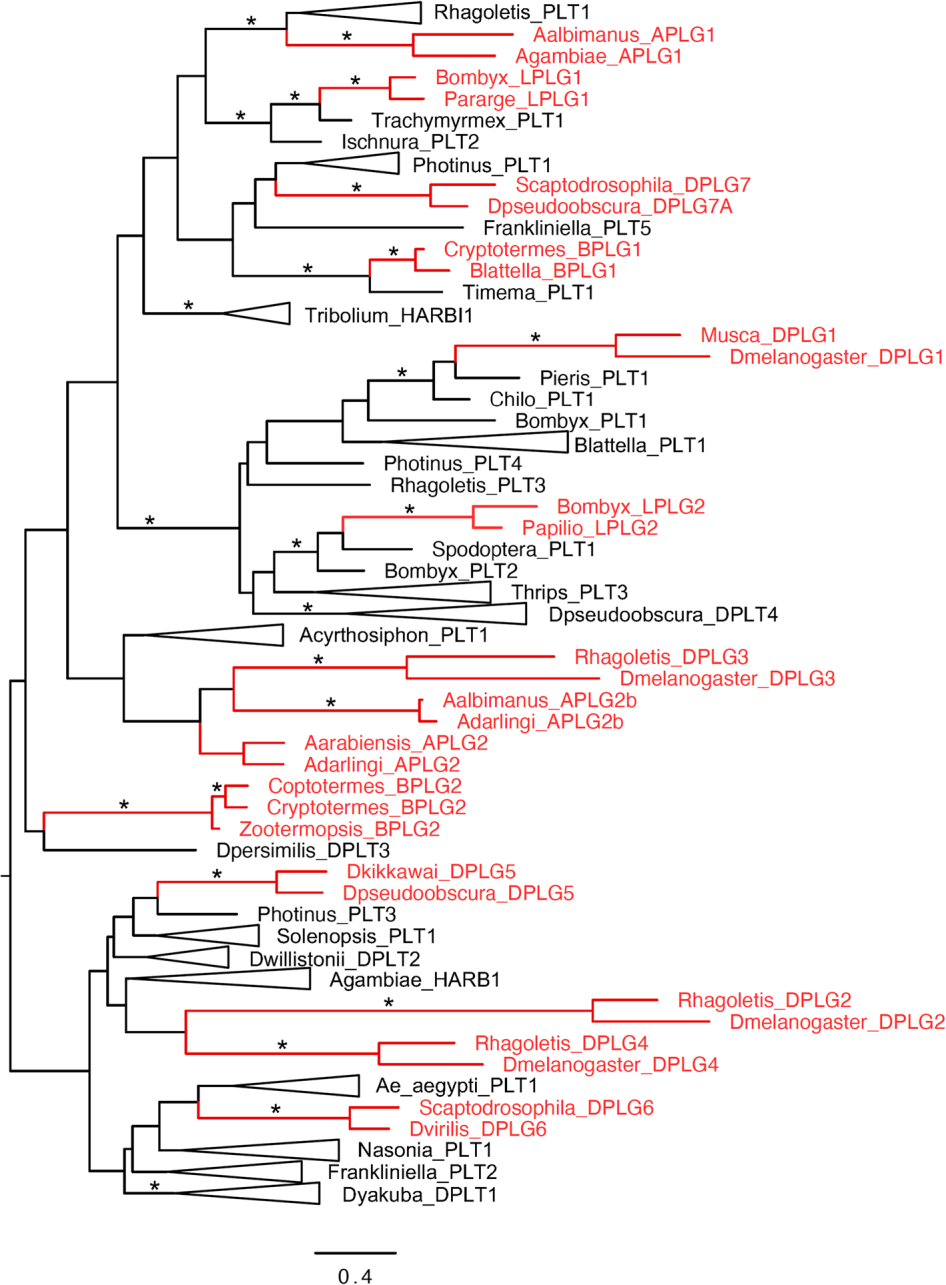
Phylogenetic relationships of transposase proteins from domesticated genes (red) and transposons (black). Collapsed clades are named by one representative transposon. Asterisks indicate nodes with bootstrap values higher than 80%. The complete phylogeny is available as Supplementary Fig. 3

As we retrieved the domestication cases above, we collected *PIF* transposases sequences from those genomes (Supplementary file 2). The phylogenetic reconstruction of transposase evolution from 90 insect *PIF*-like sequence revealed that domesticated elements are scattered throughout the *PIF* transposon transposases tree (Fig. 5; Supplementary Fig. 3; Supplementary file 3). This phylogeny suggests that the majority of the fourteen domesticated *PIF* genes found in insects evolved independently from distantly related lineages of transposons. Nodes ancestral to each *PIF*-like gene (*PLG*) are statistically well supported with the sole exception of *APLG2*. This phylogeny of *PIF*-like transposases shows that *APLG2* and *APLG2b* form a clade with low statistical support together with *DPLG3* genes (Fig. 5; Supplementary Table 4), but *APLG2* and *APLG2b* are not monophyletic within this clade. No synteny conservation was found for *APLG2* and *DPLG3* in * Anopheles* and *Drosophila*, further supporting that these two genes represent independent domestication events. So, while some *PLGs* appear to form clades, these are not well supported in agreement with the scenario of multiple independent domestication events. This is also the case for the pair of tandemly arranged genes *APLG2* and *APLG2b*. Although *APLG2b* is likely to have evolved from duplication of its neighbor gene, it might have experienced a high level of substitution, obfuscating the evolutionary relations between the two. This is supported by the length of the branch leading to *APLG2b* proteins (Fig. 5).

The genes *APLG1*, *LPLG1*, *BPLG1*, *DPLG1* and *LPLG2* form monophyletic clades with one or multiple *PIF* transposon transposase lineages, further reinforcing the hypothesis that insect *PLGs* largely evolved independently. *APLG1* is sister to a group of TEs found in dipterans, beetles and hymenopterans (Fig. 5). *LPLG1* forms a clade with TEs from ants and dragonflies, whereas *BPLG1* is sister with a stick insect element. Both *DPLG1* and *LPLG2* grouped with different TE lineages from lepidopterans.

Overall, no *PLGs* can be traced to *PIF* elements currently present in the same genomes, as already suggested for the seven *Drosophila PLGs* [13]. This lends further support to the view that insect *PLGs* represent ancient domestication events from TE lineages that have gone extinct in the species harboring those genes.

### A step-by-step model of *PIF* TE genes co-domestication

Building upon our previous work in *Drosophila* [13], we have performed exhaustive analyses of *PIF*-like sequences in the most diverse animal taxon to determine the frequency of transposase and MADF genes co-domestication (Fig. 1). We established that co-domestication events from the same TE insertion are relatively common in insects, with a minimum of four cases found collectively: *Drosophila DPLG7-DPM7* (65 Mya), *Anopheles* (*APLG1-APM1* ~ 28 Mya, and *APLG2-APM2* ~ 100 Mya), and Lepidoptera (*LPLG2-LPM2* ~ 140 Mya). Although six cases of TPase gene-only domestications were documented in *Drosophila* in our previous work (Casola, et al. 2007), we found only three additional such events in other insects (Fig. 1). While the possibility exists that some domesticated events could not be traced due to incomplete gene annotation outside *Drosophila*, these findings indicate that co-domestication occurs at least as often as the recruitment of TPase genes-only in non-*Drosophila* lineages. It is also possible that genes encoding MADF-like proteins tend to be co-domesticated in most cases but are domesticated from independent insertions or subsequently relocated to other genomic regions in some lineages (See MADF genomic distribution in Fig. 1). Indeed, we observed that in the mosquito *APLG2-APM2* and the lepidopteran *LPLG2-LPM2* pairs the two genes are proximal to each other in many species while they are farther apart in the genome of other species (Figs. 2 and 3). The growth of the intergenic region between the *PLGs* and the MADF encoding gene is the most parsimonious explanation to this pattern. Since the two genes do not need to be linked chromosomally for the co-domestication after a TE invasion, our observations speak to the specificity of the interaction and co-domestication of transposase gene and MADF/Myb-like gene from the same *PIF* TE insertion.

An observation further supporting the frequent co-domestication scenario is that in most *PIF* TE co-domestications only one of the two proteins show a predicted nuclear localization signal (NLS; Supplementary Table 5), suggesting that the two *PIF* proteins need to interact to relocate to the nucleus, as shown in rice (Hancock et al., 2010) and zebrafish (Sinzelle et al., 2008). Indeed, among co-domestication cases, we found that often only one protein, either the transposase or the MADF protein but not both, contained a predicted NLS in 33/42 species (Supplementary Table 5).

**Fig. 6:**
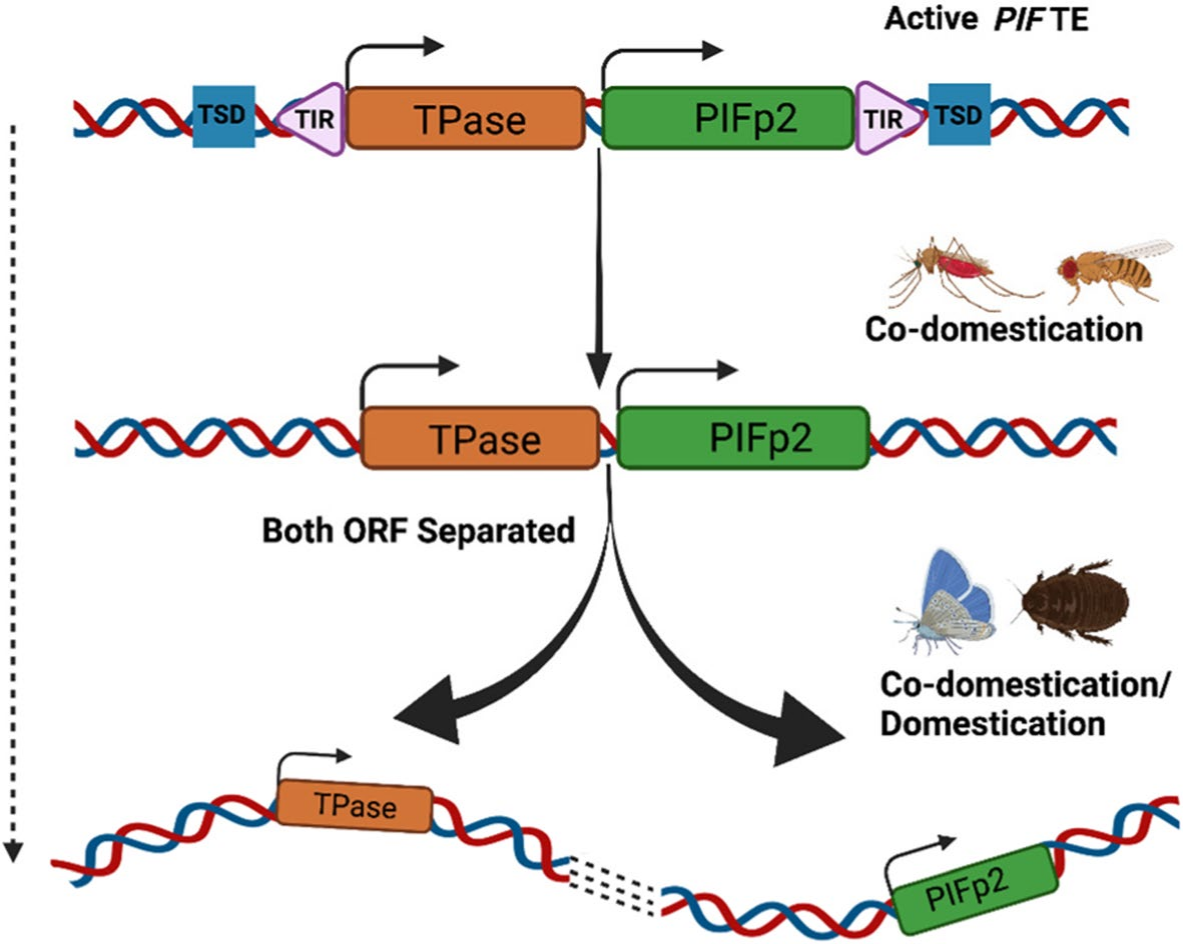
Representation of *PIF* TE genes co-domestication in a step-by-step model. We observe that transposases are often co-domesticated with the second *PIF* TE ORF from the same TE insertion but might eventually be separated in the genome

Such interaction can occur regardless of the proximity of the transposase and MADF genes. However, additional support for the requirement of co-domestication from the same TE insertion comes from the fact that *APLG1* and *APM1* are annotated as dicistronic in *A. gambiae*. Thus, we propose a step-by-step model wherein co-domestication of both *PIF* genes from the same insertion is common and is occasionally followed by the separation or movement of one of the two genes to different genomic locations, from which they might continue to interact (Fig. 6).

## Conclusions

A growing number of studies suggest that TE domestication is a common pathway for the emergence of new genes and functions. Interestingly, most of the domesticated TE genes are derived from transposases encoded by DNA transposons, although TEs of this class are typically less abundant than class I elements in eukaryotic genomes [21]. Here, we report at least six cases of domestication and co-domestication events from *PIF* TE genes in insects and one likely duplication. Those need to be added to the domestication in *Drosophila* and given our conservative approach of searching for and identifying such events, we suspect that many more are likely to have occurred during insect evolution. *PLGs* are scattered across the phylogeny of *PIF*-like sequences, supporting independence of domestication events. Moreover, we provide a step-by-step model where we show that co-domestications often occur from domesticated genes from the same insertion that subsequently separate and acquired different genomic positions, but most probably encode for proteins that continue to interact with each other. It seems that as *PIF* TEs are horizontally transferred to insect genomes there is a steady flow of *PIF*-like gene domestications, which according to the current data are likely to encode for regulatory proteins in the host genomes [28–30].

## Methods

### Database searches

**Fig. 7:**
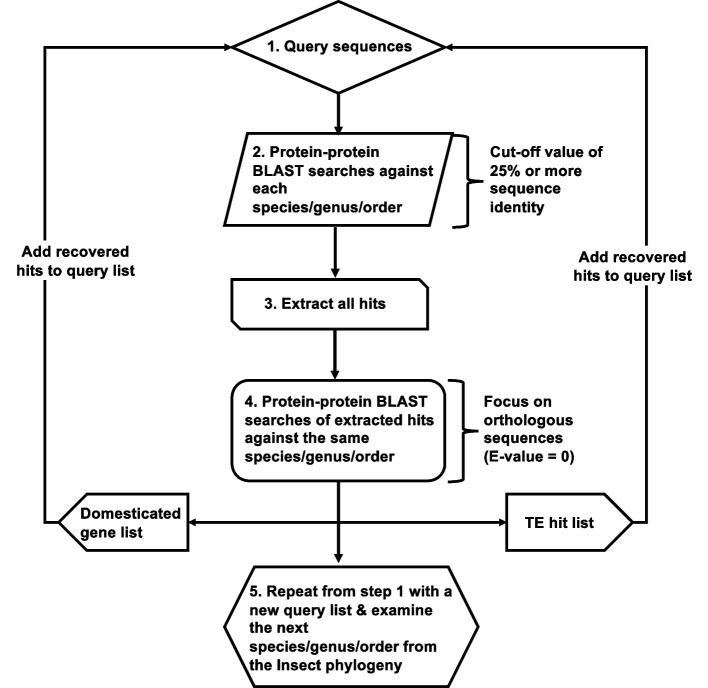
Schematic representation of the pipeline we used for the genomic data extraction and analyses of transposase hits

*PIF*-like sequences and candidate domesticated genes in non-*Drosophila* insects were identified through searches against NT, NR and some WGS NCBI databases via BLASTp and tBLASTn [41]. Searches were conducted between April 2020 and February 2022. All parameters were left to default settings. As an initial step, *Drosophila DPLG1–7* nucleotide and protein *PIF* transposase domesticated sequences, as well as previously identified *Drosophila, Anopheles,* and *Tribolium PIF*-like TEs [13] were used as queries for searches within the *Anopheles* genomes (See Supplementary Table 1 for details of the available *Anopheles* genomes). All *Anopheles* hits including TEs with 25% sequence identity or more were retained and examined for the presence of conserved transposase domain using the NCBI CDD server [42, 43]. Hits lacking functional transposase domain were discarded from the dataset. The remaining sequences were screened again against the available *Anopheles* genomes via BLASTp searches to identify orthologous sequences in at least three or more species. Only hits with an expected value of 0 in that genome database (i.e., *E* = 0) were considered for further evaluation. The presence of MADF domains in proteins flanking the *PIF* transposase hit was determined using the NCBI CDD server [42, 43]. Sequences that fitted a list of criteria (See below) were then classified as *PIF* transposase gene domestication or co-domestications and the remaining of sequences as TEs. Both groups of *PIF* transposase hits were then added to the initial query list and were used for BLASTp searches against the next insect order closely related to *Anopheles* with available genomic data. See a depiction of all the steps of this pipeline in Fig. 7. Those steps were repeated as we screened genomes across the whole insect phylogeny resulting in the evaluation of more than 250 queries. Once all insect orders were screened the complete list of 250 queries was used again in a second round of screening within the *Anopheles* and Lepidoptera genomes. This approach and the orthologous assignment approach described below also produced the most up to date dating of the domestication of *DPLG1–7* (55–192 Mya; http://www.timetree.org; Supplementary Table 6).

We screened all species with sequenced and annotated genomes within (in alphabetic order): Archaeognatha, Blattodea, Coleoptera, Collembola, Dermaptera, Diplura, Diptera, Ephemeroptera, Hymenoptera, Lepidoptera, Megaloptera, Odonata, Orthoptera, Phasmatodea, Plecoptera, Psocodea, Siphonaptera, and Thysanoptera. We couldn’t screen (in alphabetic order): Embioptera, Grylloblattodea, Mantodea, Mantophasmatodea, Mecoptera, Neuroptera, Protura, Raphidioptera, Zoraptera and Zygentoma (Thermobia) because of lack of sequenced and annotated genomes. See Supplementary Table 1.

We found domestications events within:

- *Anopheles* (order Diptera): *Anopheles albimanus, Anopheles arabiensis, Anopheles bwambae, Anopheles coluzzi, Anopheles crystyi, Anopheles darlingi, Anopheles gambiae strain PEST, Anopheles melas, Anopheles merus, Anopheles quadriannulatus, Anopheles sinensis,* and *Anopheles stephensi.*
- Lepidoptera: *Amyelois transitella, Arctia plantaginis, Bicyclus anynana, Bombyx mandarina, Bombyx mori, Chilo suppressalis, Danaus plexippus plexippus, Galleria mellonella, Helicoverpa armigera, Heliothis virescens, Hyposmocoma kahamanoa, Leptidea sinapis, Manduca sexta, Maniola hyperantus, Operophtera brumata, Ostrinia furnacalis, Papilio bianor, Papilio dardanus Tibullus, Papilio glaucus, Papilio machaon, Papilio Memnon, Papilio polytes, Papilio xuthus, Pararge aegeria, Pieris rapae, Plutella xylostella, Spodoptera exigua, Spodoptera frugiperda, Spodoptera litura, Trichoplusia ni, Vanessa tameamea,* and *Zerene cesonia.*
- Blattodea: *Blattella germanica, Coptotermes formosanus, Cryptotermes secundus, Nasutitermes exitiosus, Periplaneta americana,* and *Zootermopsis nevadensis.*

Note that we are using the annotations as provided by the genome producers and have not attempted to manually annotate of all the genes we found.

### Structural and sequence features that support domestication or co-domestication and transposable element identification

As described above, only hits of potentially domesticated transposases with an expected value of *E* = 0 to the genomes of closely related species were considered for further evaluation, i.e., were assumed to be conserved functional genome proteins derived from *PIF* TEs. To confirm that these hits were domestications or co-domestications and not *PIF* TEs, we checked for specific features. In brief, we examined the region for an intact coding region, conservation of intron-exon structure across the independent species hits and the absence of TE hallmarks (TIRs and TSD). The sequence of each retrieved PIF-like element was extended to add the 3000 bp flanking regions of both 5′ and the 3′ ends of the element using the genome assembly sequence. TIRs were searched via BLASTn analyses of the entire PIF-like element and its flanking regions against itself (self-BLAST). TIRs in *PIF* TEs are relatively short (~ 15 bp) but can be readily identified in self-homology searches. For sequences with apparent TIRs, we inspected the presence of a target site duplication, which is typically 3-bp long in *PIF* transposons [8]. Hits with sequence similarity or clustering phylogenetically with known *PIF* TEs were also retained (See phylogenetic relationships in Results and Discussion). Co-domestications were annotated if a protein with a MADF domain was found next to the domesticated transposase. This is because MADF proteins evolve fast [28, 29] and *PIF* TE gene 2 cannot easily be detected by BLAST. Furthermore, we determined if those putative genes were under purifying selection and had conserved synteny across the species where that domestication or co-domestication was found, as described below.

### Orthology assignment and gene structure of domesticated genes

Orthology of domesticated genes was established by examining the microsynteny of flanking genes. Genes were considered orthologs when the microsynteny was conserved on both sides of the gene in closely related species and on at least one side of the gene in more distantly related genera. For genomes that were sequenced but not annotated, we used the Graphics track option of tBLASTn in NCBI genome database to collect the complete sequence of the hits and the exon-intron structure of each hit was analyzed and annotated manually using the ExPASy bioinformatics resource portal [44] and refined by multialignments with orthologous genes.

Then tBLASTn searches using newly discovered domesticated proteins against *Anopheles*, Lepidoptera, and Blattodea genomes were used to identify scaffolds containing the possible orthologs. The scaffolds were retrieved from genome assemblies (Supplementary Table 1) and were first manually inspected using the NCBI conserved domain database [42, 43]. We compared approximately 10 kb upstream and downstream regions flanking each domesticated gene for synteny conservation, thus allowing us to confirm if detected sequences are orthologous.

### Sequence evolution and function of domesticated *PIF* genes

To study the mode of evolution of domesticated genes, the ratio of nonsynonymous substitutions per nonsynonymous site to synonymous substitutions per synonymous site (dN/dS) was estimated using the CodeML algorithm implemented in EasyCodeML [45]. We used branch models with a null model assuming that all lineages are evolving at the same rate (one-ratio model) and an alternative model in which the dN/dS ratio for all lineages was fixed to 1. The likelihood ratio test (LRT [46]) was conducted to compare these models. *P*-values of 0.05 or smaller in the LRTs were considered to be statistically significant and often indicated that domesticated genes are evolving under purifying selection (dN/dS < 1 in the one-ratio model) and not accumulating changes at random as would be expected of TE insertions.

We used NLS mapper to predict nuclear localization signals in the domesticated proteins (https://nls-mapper.iab.keio.ac.jp/cgi-bin/NLS_Mapper_form.cgi).

### Expression of domesticated *PIF* genes

Evidence of transcription of domesticated *PIF* genes in *Anopheles gambiae* and *Bombyx mori* was retrieved from previous publications [47, 48] using the gene annotations. Robust multi-array average (RMA) and transcripts per kilobase million (TPM) expression values were retrieved for the adult tissues analyzed in those references.

### Protein alignments and phylogenetic relationships

All insect transposase sequences from both domesticated genes and *PIF* transposons obtained from databases, published sequences and BLAST searches were aligned using MAFFT v7 with default settings [49] and inspected to identify and remove sequences with large gaps in conserved blocks. After removing sites with gaps in the remaining proteins, alignments were used to infer phylogenetic relationships using PhyML v3.0 with default settings except for selecting the LG substitution model [50]. Preliminary phylogenies were carried out using the PhyML SH-like approximate likelihood-ratio test to assess node support and identify transposase groups with at least 0.9/1 support, which were considered candidate monophyletic clades. Individual sequences outside of clades were used as queries for tBLASTn searches against whole-genome sequences from insects, in order to retrieve possible closely related sequences and obtain new clades. After exhausting all phylogenetically isolated transposases, a final tree was built with PhyML and a statistical branch support obtained using 100 bootstrap replicates. Phylogenies were visualized and edited using FigTree v1.4.4 (http://tree.bio.ed.ac.uk/software/figtree) and Inkscape (https://inkscape.org).

## Supplementary Information

**Additional file 1 Supplementary Fig. 1.** Syntenic relationship depicted for every case of domestication and domestication*.* Two species were chosen based on the distant phylogenetic relationship. A) *Anopheles* co-domestication case 1 (*APLG1* & *APM1*). B) *Anopheles* co-domestication case 2 (*APLG2* & *APM2).* C) *Lepidoptera* domestication case 1 (*LPLG1 &LPM1*). D) *Lepidoptera* domestication & co-domestication case 2 (*LPLG2 &LPM2*). E) *Blattodea* domestication case 1 (*BPLG1*). F) *Blattodea* domestication case 2 (*BPLG2*).

**Additional file 2 Supplementary Fig. 2.** *APLG1* and *APM1* in *A. gambiae* are annotated in a single transcript, *AGAP029479*-RA, in VectorBase.

**Additional file 3 Supplementary Fig. 3.** Complete phylogenetic relationships of TPase proteins from domesticated genes (red) and transposons (black). Collapsed clades are named by one representative transposon. Asterisks indicate nodes with bootstrap values higher than 80%.

**Additional file 4 Supplementary file 1.** Protein sequences of all the domesticated proteins described in this work. Predicted NLS are highlighted in yellow.

**Additional file 5 Supplementary file 2.** Protein sequences of all *PIF* TE transposases, domesticated or not, used in Fig. 5.

**Additional file 6 Supplementary file 3.** Alignment in fasta format of all protein sequences of all *PIF* TE transposases, domesticated or not, used in Fig. 5.

**Additional file 7 Supplementary Table 1.** Information about the insect genomes explored and *PIF* TE domestications inferred.

**Additional file 8 Supplementary Table 2.** dN/dS ratio selection regime estimates for the *PIF* TE domesticated proteins.

**Additional file 9 Supplementary Table 3.** Expression data for the *PIF* TE domesticated proteins.

**Additional file 10 Supplementary Table 4.** Information about all the protein sequences of all *PIF* TE transposases, domesticated or not, used in Fig. 5.

**Additional file 11 Supplementary Table 5.** Presence/absence of predicted nuclear localization signal in newly described *PIF* TE domesticated proteins.

**Additional file 12 Supplementary Table 6.** Details of genomes and presence/absence of *DPLG1–7* in insect genomes.
