# Supplementary material for: Recurrent co-domestication of PIF/Harbinger transposable element proteins in insects
Source: Mob DNA. 2022 Nov 30;13:28. doi: 10.1186/s13100-022-00282-2 (PMC9710019; doi:10.1186/s13100-022-00282-2)
Supplement: Supplementary file 1 — Additional file 1 Supplementary Fig. 1. Syntenic relationship depicted for every case of domestication and domestication. Two species were chosen based on the distant phylogenetic relationship. A) Anopheles co-domestication case 1 (APLG1 & APM1). B) Anopheles co-domestication case 2 (APLG2 & APM2). C) Lepidoptera domestication case 1 (LPLG1 &LPM1). D) Lepidoptera domestication & co-domestication case 2 (LPLG2 &LPM2). E) Blattodea domestication case 1 (BPLG1). F) Blattodea domestication case 2 (BPLG2). [file 13100_2022_282_MOESM1_ESM.pdf]

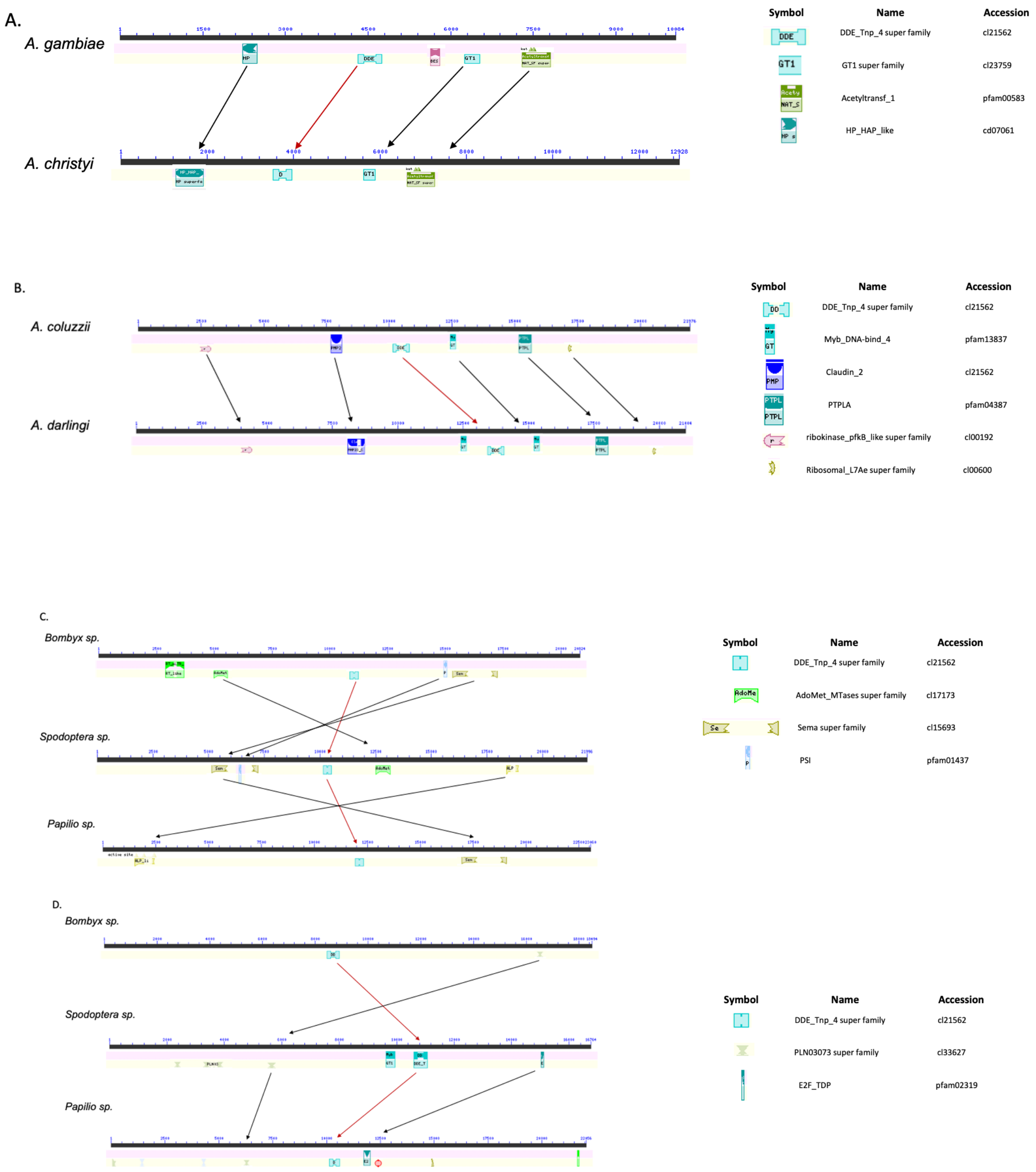

A.
A. gambiae
A. christyi
Symbol
Name
Accession
DDE_Tnp_4 super family
cl21562
GT1 super family
cl23759
Acetyltransf_1
pfam00583
HP_HAP_like
cd07061
B.
A. coluzzii
A. darlingi
Symbol
Name
Accession
DDE_Tnp_4 super family
cl21562
Myb_DNA-bind_4
pfam13837
Claudin_2
cl21562
PTPLA
pfam04387
ribokinase_pfkB_like super family
cl00192
Ribosomal_L7Ae super family
cl00600
C.
Bombyx sp.
Spodoptera sp.
Papilio sp.
Symbol
Name
Accession
DDE_Tnp_4 super family
cl21562
AdoMet_MTases super family
cl17173
Sema super family
cl15693
PSI
pfam01437
D.
Bombyx sp.
Spodoptera sp.
Papilio sp.
Symbol
Name
Accession
DDE_Tnp_4 super family
cl21562
PLN03073 super family
cl33627
E2F_TDP
pfam02319

E.

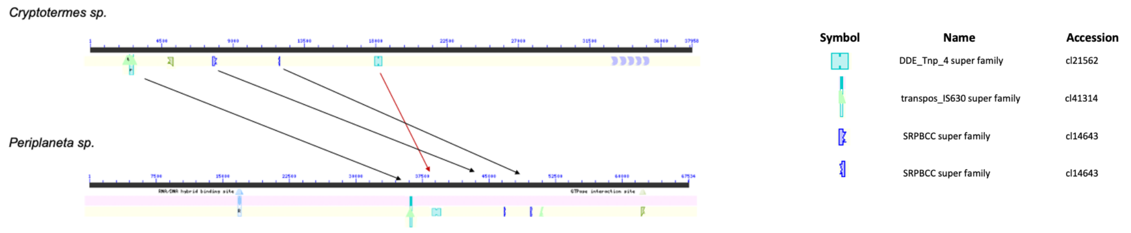

F.

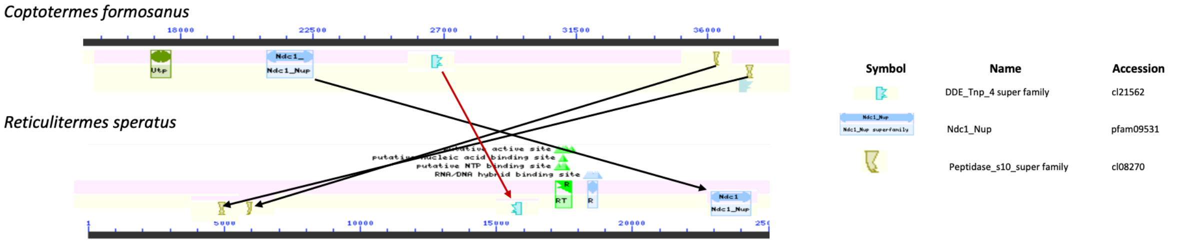

Supplementary Figure 1. Syntenic relationship depicted for each independent domestication and co-domestication case. Two distantly related species within each taxon were selected. A) *Anopheles* co-domestication case 1 (*APLG1* & *APM1*). B) *Anopheles* co-domestication case 2 (*APLG2* and *APM2).* C) *Lepidoptera* domestication case 1 (*LPLG1* and *LPM1*). D) *Lepidoptera* domestication & co-domestication case 2 (*LPLG2* and *LPM2*). E) *Blattodea* domestication case 1 (*BPLG1*). F) *Blattodea* domestication case 2 (*BPLG2*).
