# Supplementary material for: Recurrent co-domestication of PIF/Harbinger transposable element proteins in insects
Source: Mob DNA. 2022 Nov 30;13:28. doi: 10.1186/s13100-022-00282-2 (PMC9710019; doi:10.1186/s13100-022-00282-2)
Supplement: Supplementary file 2 — Additional file 2 Supplementary Fig. 2. APLG1 and APM1 in A. gambiae are annotated in a single transcript, AGAP029479-RA, in VectorBase. [file 13100_2022_282_MOESM2_ESM.pdf]

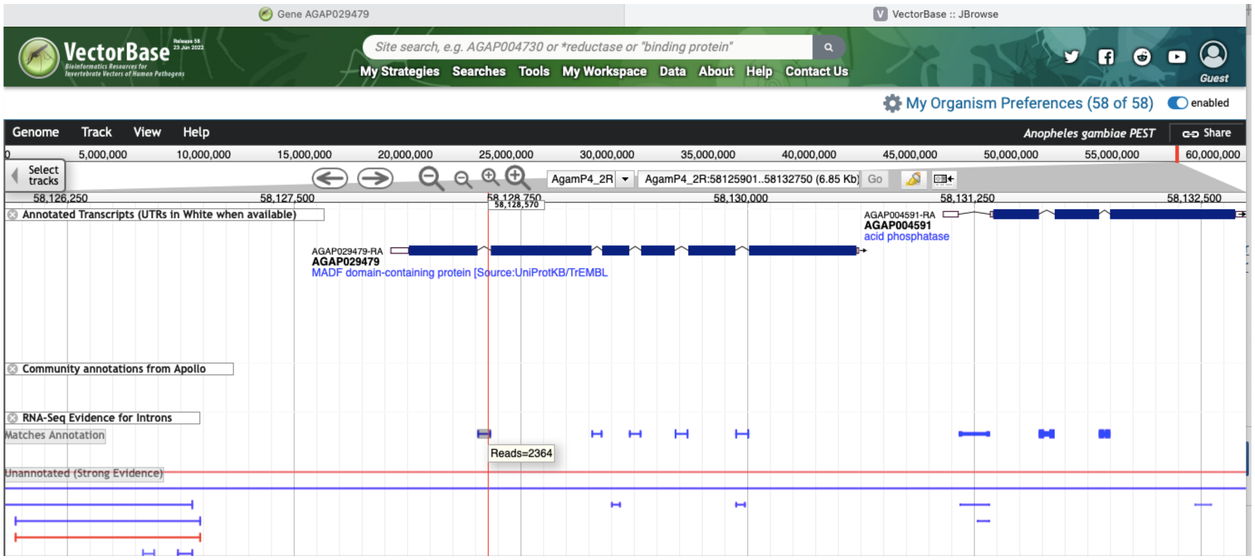

Supplementary Figure 2. *APLG1* and *APM1* in *A. gambiae* are annotated in a single transcript, *AGAP029479*-RA, in VectorBase. Reads supporting the first intron are shown (i.e., 2364 reads). Reads supporting other introns are 2024, 1424, 1791 and 2139.
