# Supplementary material for: Recurrent co-domestication of PIF/Harbinger transposable element proteins in insects
Source: Mob DNA. 2022 Nov 30;13:28. doi: 10.1186/s13100-022-00282-2 (PMC9710019; doi:10.1186/s13100-022-00282-2)
Supplement: Supplementary file 3 — Additional file 3 Supplementary Fig. 3. Complete phylogenetic relationships of TPase proteins from domesticated genes (red) and transposons (black). Collapsed clades are named by one representative transposon. Asterisks indicate nodes with bootstrap values higher than 80%. [file 13100_2022_282_MOESM3_ESM.pdf]

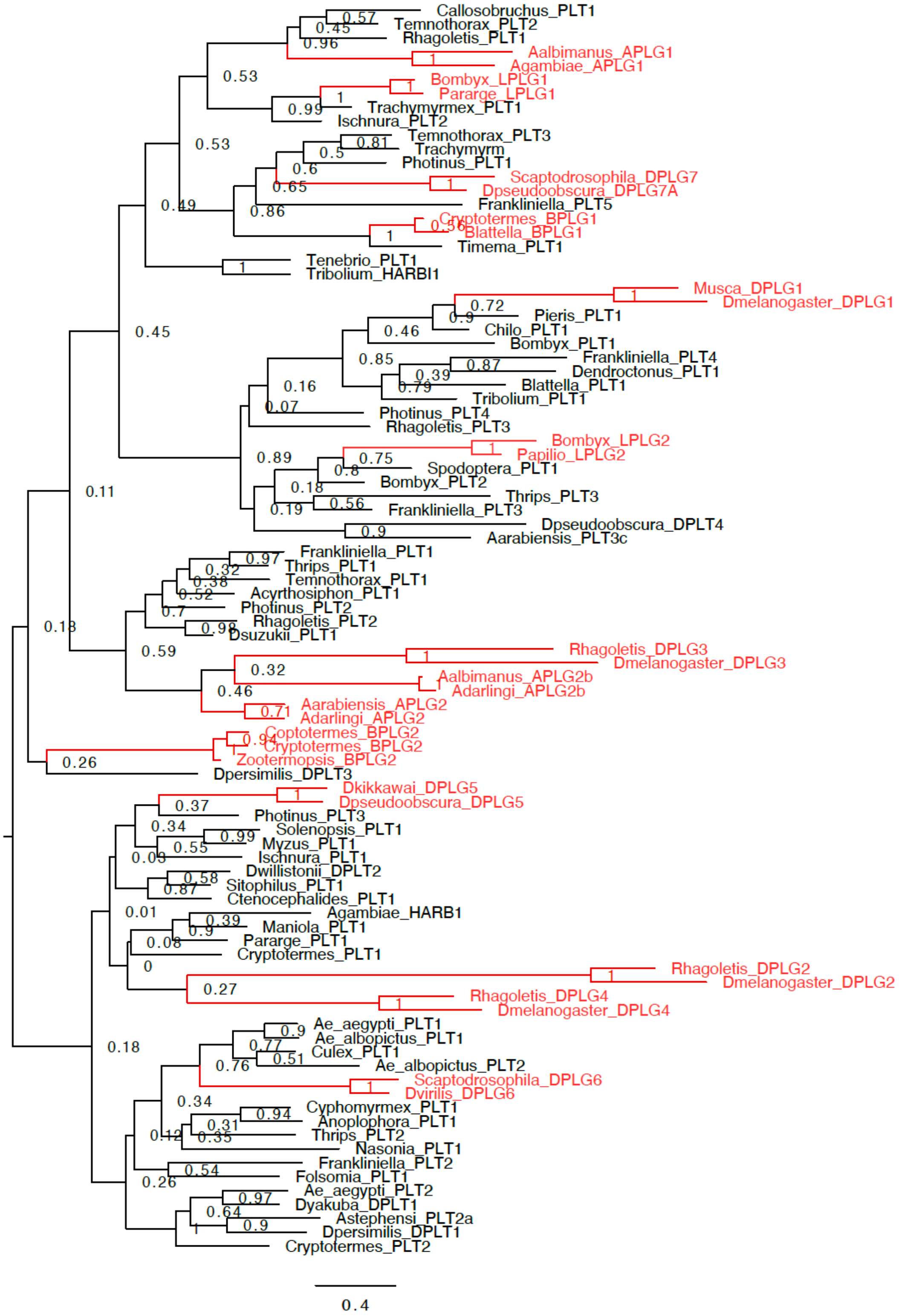

Supplementary Figure 3. Complete phylogenetic relationships of TPase proteins from domesticated genes (red) and transposons (black). Collapsed clades are named by one representative transposon. Asterisks indicate nodes with bootstrap values higher than 80%.
