# Supplementary material for: Recurrent co-domestication of PIF/Harbinger transposable element proteins in insects
Source: Mob DNA. 2022 Nov 30;13:28. doi: 10.1186/s13100-022-00282-2 (PMC9710019; doi:10.1186/s13100-022-00282-2)
Supplement: Supplementary file 4 — Additional file 4 Supplementary file 1. Protein sequences of all the domesticated proteins described in this work. Predicted NLS are highlighted in yellow. [file 13100_2022_282_MOESM4_ESM.docx]

**Supplementary file 1.** Protein sequences of all the domesticated proteins described in this

work. Predicted NLS are highlighted in yellow.

**APLG1 protein sequences in *Anopheles***

>A_gambiae_APLG1

MHFSHYIRNVVPKYSDEEFLRQFRIGRKAVQMICNHLETTSAYKKLRGHGGYEAISPQTHVLSFLWFLGHDKTSYRDVATQFNLSVSCLHSVICRVADAILSMKHILMIPLSEARKTASDIAFSKKCNFSGVIGCVGGTQIKIDKPRENPERYLLTTGHYSIQLQAIIDENLRFVDVFVEYPDEPELLETVKRICGNRYCLLGNASYPCMNQLLVPYPVDEVLLTQAQKTYNDHLQTVTSQCNQIFAHLKTRFRRLHHLKGRHLSRMVDLIKVCCILHNLASVAELKQLEGDMNQLPPEPIFTYISETRAHVDIVHGQQKRDKICAKYQ

> A_coluzzii_APLG1

MHFSHYIRNVVPKYSDEEFLRQFRIGRKAVQMICNHLETTSAYKKLRGHGGYEAISPQTHVLSFLWFLGHDKTSYRDVATQFNLSVSCLHSVICRVADAILSMKPILMIPLSEARKTASDIAFSKKCNFSGVIGCVGGTQIKIDKPRENPERYLLTKGHYSIQLQAIIDENLRFVDVFVEYPDEPELLETVKRICGNRYCLLGNASYPCMNQLLVPYPVDEVLLTQAQKTYNDHLQTVTSQCNQIFAHLKTRFRRLHHLKGRHLSRMVDLIKVCCILHNLASVAELKQLEGDMNQLPPEPIFTYISETRAHVDIVHGQQKRDKICAKYQ

>A_bwambae_APLG1

MHFSHYIRNVVPKYSDEEFLRQFRIGRKAVQMICNHLETTSAYKKLRGHGGYEAISPQTHVLSFLWFLGHDKTSYRDVATQFNLSVSCLHSVICRVADAILSMKHILMIPLSEARKTASDIAFSKKCNFSGVIGCVGGTQIKIDKPRENPERYLLTKGHYSIQLQAIIDENLRFVDVFVEYPDEPELLETVKRICGNRYCLLGNASYPCMNQLLVPYPVDEVLLTQAQKTYNDHLQTVTSQCNQIFVHLKTRFRRLHHLKGRHLARMVDLIKVCCILHNLASVAELKQLEGDMNQLPPEPIFTYISETRAHVDIVHGQQKRDKICAKYQ

>A_arabiensis_APLG1

MHFSHYIRNVVPKYSDEEFLRQFRIGRKAVQMICNHLETTSAYKKLRGHGGYEAISPQTHVLSFLWFLGHDKTSYRDVATQFNLSVSCLHSVICRVADAILSMKHILMIPLSEARKTASDIAFSKKCNFSGVIGCVGGTQIKIDKPRETPERYLLTKGHYSIQLQAIIDENLRFVDVFVEYPDEPELLETVKRICGNRYCLLGNASYPCMNQLLVPYPVDEVLLTQAQKTYNDHLQTVTSQCNQIFAHLKTRFRRLHHLKGRHLSRMVDLIKVCCILHNLASVAELKHLEGDMNQLPPEPIFTYISETRAHVDIVHGQQKRDKICAKYQ

> A_merus_APLG1

MHFSHYIRNVVPKYSDEEFLRQFRIGRKAVQMICNHLETTSAYKKLRGHGGYEAISPQTHVLSFLWFLGHDKTSYRDVASQFNLSVSCLHSVICRVADAILSMKHILMISLSEARKTASDIAFSKKCNFSGVIGCVGGTQIKIDKPRENPERYLLTKGHYSIQLQAIIDENLRFVDVFVEYPDEPELLETVKRICGNRYCLLGNASYPCMSQLLVPYPVDEVLLTQAQKTYNDHLQTVTSQCNQIFAHLKTRFRRLHHLKGRHLSRMVDLIKVCCILHNLASVAELKQLEGDVNQLPPEPIFTYISETRAHEDIVHGQQKRDKICAKYQ

>A_quadriannulatus_APLG1

MHFSHYIRNVVPKYSDEEFLRQFRIGRKAVQMICNHLETTSAYKKLRGHGGYEAISPQTHVLSFLWFLGHDKTSYRDVATQFNLSVSCLHSVICRVADAILSMKHILMIPLSEAGKTASDIAFSKKCNFSGVIGCVGGTQIKIDKPRENPDRYLLTKGHYSIQLQAIIDENLRFVDVFVEYPDEPELLETVKRICGNRYCLLGNASYPCMNQLLVPYPVDEVLLTQAQKTYNDHLQTVTSQCNQIFAHLKTRFRRLHHLKGRHLSRMVDLIKVSCILHNLASVEELKQLEGDMNQLHPEPIFTYISETRAHVDIEHGQQKRDKICAKYL

>A_melas_APLG1

MHFSHYIRNVVPKYSDEEFLRQFRIGRKAVQMICNHLETTSVYKKLRGHGGYEAISPQTHVLSFLWFLGHDKTSYRDVATQFNLSVSCLHSVICRVADAILSMKHILMIPLSEARKTASDIAFSKKCNFSGVIGCVGGTQIKIDKPRENPERYLLTKGHYSIQLQAIIDENLRFFDVFVEYPDEPELLETVKRICGNRYCLLGNASYPCMNQLLVPYPVDEVLLTQAQKAYNDHLQTVTSQCNQIFAHLKTRFRRLHHLKGRHLARMVDLIKVCCILHNLASVEELKQLEGDMNQLPPEPIFTYISETRAHVDIEHGQQKRDKICAKYL

>A_christyi_APLG1

MHFSHYMQNVVPDYSDEEFLRNFRMGRNAVQIVCSHLETTSAYKKLKGHGGYEAISPQTHVLAFLWFLGHDKTSYRDVATQFNLSVSCLHNVICRVADSILSLKQTLMKPLTEETKGTSASVFAEMCNIFGVIGCVGGTQIKIDKPIENAEKYLLTKGHYFIQLQAIIDENLRFVDVFVEYPGEPDLVATVKDICSGNYCLLGNDSYPCLNQLLVPYSIDEAAGTLTRAQKTYNEQLLYVTDQCNQIFAHLKTRFRRLYHLKGRHLSRVVSLIKVCCILHNLASPEEMKLLERNEKELPSEPIFNYAYENHSQGEILLGRQKRDRICANYV

**APLG2 protein sequences in *Anopheles***

>A_gambiae_APLG2

MDNAHKRQVVENLFLTFATKQAELFKQYDANRKKRSKIFLQRLRYQLKRNGENHRKEWSSKLEAELLESIPTRKIWKLSRNDKWFRELFEQEDNEDDLLRYFRMGRAMYDTLVAMLRQDLAPHPLLAAQSCSAEKKVGIGLYKLTTGADYTTIGNMFGVHKATVKNCVHQFCKSLVKNYMDSEIYLPDQEEMAEISKSFEDKSDIPLVIGAMGRLHVPITPSLADSKNYINGRKWPSLILQAVVDNNHCFRHITCGHVGATEDSVVLGDSGLYQHFDGADLPSQNINGNTVKSFIVSDTVYPLLPWLQHGYMTPTTTEEETFNEHLNKARVAVDEAFDRLRARFRILQRKIDIDINFVPQILLTCCILHNFLEKNKTPFLETWSEALQESNQKYPQPDGSASLNYTTTVEGESIRDVLKEHLHSKYVLFRTIEYNQVYFIGEGNN

>A_coluzii_APLG2

MDNAHKRQVVENLFLTFATKQAELFKQYDANRKKRSKIFLQRLRYQLKRNGENHRKEWSSKLEAELLESIPTRKIWKLSRNDKWFRELFEQEDNEDDLLRYFRMGRAMYDTLVAMLRQDLAPHPLLAAQSCSAEKKVGIGLYKLTTGADYTTIGNMFGVHKATVKNCVHQFCKSLVKNYMDSEIYLPDQEEMAEISKSFEDKSDIPLVIGAMGRLHVPITPSLADSKNYINGRKWPSLILQAVVDNNHCFRHITCGHVGATEDSVVLGDSGLYQHFDGADLPSQNINGNTVKSFIVSDTVYPLLPWLQHGYMTPTTTEEETFNEHLNKARVAVDEAFDRLRARFRILQRKIDIDINFVPQILLTCCILHNFLEKNKTPFLETWSEALQESNQKYPQPDGSASLNYTTTVEGESIRDVLKEHLHSKYVLFRTIEYNQVYFIGEGNN

>A_arabiensis_APLG2

MDNAHKRQVVENLFLTFATKQAELFKQYDANRKKRSKIFLQRLRYQLKRNGENHRKEWSSKLEAELLESIPTRKIWKLSRNDKWFRELFEQEDNEDDLLRYFRMGRAMYDTLVAMLRQDLAPHPLLAAQSCSAEKKVGIGLYKLTTGADYTTIGNMFGVHKATVKNCVHQFCKSMVKNYMDSEIYLPDQEEMAEISKSFEDKSDIPLVIGAMGRLHVPITPSLADSKNYINGRKWPSLILQAVVDNNHCFRHITCGHVGATEDSVVLGDSGLYQHFDGADLPSQNINGNTVKSFIVSDTVYPLLPWLQHGYMTPTTTEEETFNEHLNKARVAVDEAFDRLRARFRILQRKIDIDINFVPQILLTCCILHNFLEKNKTPFLEAWSEALQESNQRYPQPDGSASLNYTTTVEGETIRDVLKEHLHSKYVLFRTIEYNQVYFIGEGNN

>A_stephensi_APLG2

MDPGHKRQMVENLFLTFATKQAELFKQYDANRKKRSKIFLQRLRYQLKRNGESHRKEWSSRLESELLDSIPTRKIWKLSRNDRWFRELFQHENNDQELLHHFRMDRSMFDTLVAMLCQDLAPHPLLAAQSCSTEKKVGIGLYKLTTGADYATIGNLFGVHKATVKNCVHQFCKSMVKNYMDTEIYLPDQEEMGEISKAFEDRSDIPMIIGAMGRLHVPITPSLADSKNYINARKWPSLILQAVVDNNHCFRHITCGHVGATEDSVVLGDSGLYQHFDGAELPTQSINGTTVKSFVVSESAYPLLPWLQHGYVTPQTTEEETFNEHLNKARICVDEAFEKLRVRFRILQRKIDIDINFVPQILLTCCILHNILEKNQTPYLDEWKEALLELRGKYPQPDGAASTKYTTTVEGESLRDVLKEHLQSKYVLYRTIEYNQVYFINEGTN

>A_sinensis_APLG2

MEQEQKRQIIENLFTSYAAKRAELVRQYETNRKKRSKIFLQRLRYQLKRNSEAHRSEWCSTARTVVLDSIPTRKIWKLNRCDKWFREVYEAEGNEAAMLESFRMDKDTYEVLVDALTNDLAPHPLLAAQSCSTEKKVGIGLYKLTSGADYATIGNLFGVHKATVKNCVHQFCKAMVKHFMDVEIFLPNSDEMAEISKSFEEKCDIPLIIGAMGRTHIPITPSAADSKNYANAKKWPSLILQAVVDNNHLFRHITCGHVGCTEEAAVLGDSGLYQHFSNVEMLCQSINENTVKSFIVTDPPYPLLPWLMHGYTGMSLSSEEETFNEHLAKAKIVVDEAFMKLRARFRILQRKIDIDINFVPQILLTCCILHNLLEKRQVPFCDEWTDLLRDSEQKYPQPDGSSATSYTTTVEGEAARDILKEYVQSKYMLYRSIDYGHVYFISGEN

>A_darlingi_APLG2

MEDDEEMKRIAIQTTFSAYAAKQVELAKQYETNRKKRCKIFLQRLRYQLKRNGEQHQKEWSEILQEQLLDSVPTRKFWKLNRSDQWYRNLFEGEEDNADQLFENFRLDRPTYDMLVEALNPDMAPHPLLISQSCSTEKKVAVALYKLISGSDYASVGDQFGVHKATVKNCLFQFCKALVKNFMDAEIALPLTDEAMEISSAFEEKCDLPMVMGALGLLHIPITPSGAESKNYLNSKKWASITLQAVVDHNHLFRHITCGHTGCTEESAVLTDSGLYQHFENAEMPAQMINGNPVQGYIVTEPTYPLLPWLIHDYTPLNNPITTEEQTFNDHLAKAKAAVDQAFTRLRARFGILQRKIDIDINFVPQILLTCCILHNILEKRKMKFEDEWIEQMTAADQKYPQPDGSIVTNYTTSVEGETVRDQLKDHVQSKYMLFRSIDYSQVYFINDPNH

>A_albimanus_APLG2

MEDDAEMKRIAIRNMFTAYAAKQVELSKQYDTNRKKRCKIFLQRLRYQLKRNGEQHQKEWSEILREQLLDSVPTRKFWKLNRSDQWYRNMFESEEDNADQLFEHFRLDRATYDILVEALNPDMAPHPLLVSQSCSTEKKVAVAIYKLINGSDYASVGEQFGVHKATVKNCLFQFCKAMVKHFMDAEIALPTADEALEISSAFEEKCDLPMVIGALGLLHIPITPSGSEAKNYLNSKKWSSLTLQAVVDHNHLFRHITCGHIGCTEESAVLADSGLYQHFENTEMPAQMINGNPVQGYIVTEPTYPLLPWLIHGYTPLNNALTTEEQTFNDHLAKAKAAVDQGFTRLRARFGILQRKIDIDINFVPQILLTCCILHNILEKRKIKFDDEWIEQMTAADQKYPQPDGSIVTNYTTSVEGETVRDQLKDHIQSKYMLFRSIDYSQVYFINDPNQ

>A_quadriannulatus_APLG2

MDNAHKRQVVENLFLTFATKQAELFKQYDANRKKRSKIFLQRLRYQLKRNGENHRKEWSSKLEAELLESIPTRKIWKLSRNDKWFRELFEQEDNEDDLLRYFRMGRAMYDTLVAMLRQDLAPHPLLAAQSCSAEKKVGIGLYKLTTGADYTTIGNMFGVHKATVKNCVHQFCKSMVKNYMDSEIYLPDQEEMAEISKSFEDKSDIPLVIGAMGRLHVPITPSLADSKNYINGRKWPSLILQAVVDNNHCFRHITCGHVGATEDSVVLGDSGLYQHFDGADLPSQNINGNTVKSFIVSDTVYPLLPWLQHGYMTPTTTEEETFNEHLNKARVAVDEAFDRLRARFRILQRKIDIDINFVPQILLTCCILHNFLEKNKTPFLEAWSEALQEANQKYPQPDGSASLNYTTTVEGESIRDVLKEHLHSKYVLFRTIEYNQVYFIGEGNN

>A_bwambae_APLG2

MDNAHKRQVVENLFLTFATKQAELFKQYDANRKKRSKIFLQRLRYQLKRNGENHRKEWSSKLEAELLESIPTRKIWKLSRNDKWFRELFEQEDNEDDLLRYFRMGRAMYDTLVAMLRQDLAPHPLLAAQSCSAEKKVGIGLYKLTTGADYTTIGNMFGVHKATVKNCVHQFCKSMVKNYMDSEIYLPDQEEMAEISKSFEDKSDIPLVIGAMGRLHVPITPSLADSKNYINGRKWPSLILQAVVDNNHCFRHITCGHVGATEDSVVLGDSGLYQHFDGADMPSQNINGNTVKSFIVSDTVYPLLPWLQHGYMTPTTTEEETFNEHLNKARVAVDEAFDRLRARFRILQRKIDIDINFVPQILLTCCILHNFLEKNKTPFLEAWSEALQESNQKYPQPDGSASLNYTTTVEGESIRDILKEHLHSKYVLFRTIEYNQVYFIGEGNN

>A_melas_ APLG2

MDNAHKRQVVENLFLTFATKQAELFKQYDANRKKRSKIFLQRLRYQLKRNGENHRKEWSSKLEAELLESIPTRKIWKLSRNDKWFRELFEQEDNEDDLLRYFRMGRAMYDTLVAMLRQDLAPHPLLAAQSCSAEKKVGIGLYKLTTGADYTTIGNMFGVHKATVKNCVHQFCKSMVKNYMDSEIYLPDQEEMEEISKSFEDKSDIPLVIGAMGRLHVPITPSLADSKNYINGRKWPSLILQAVVDNNHCFRHITCGHVGATEDSVVLGDSGLYQHFDGADMAFPEHQRKHGEILYRVGYGLPAAALATARLHDPDDDGGGNL

>A_merus_ APLG2

MDNAHKRQVVENLFLTFATKQAELFKQYDANRKKRSKIFLQRLRYQLKRNGENHRKEWSSKLEAELLESIPTRKIWKLSRNDKWFRELFEQEDNEDDLLRYFRMGRAMYDTLVAMLRQDLAPHPLLAAQSCSAEKKVGIGLYKLTTGADYTTIGNMFGVHKATVKNCVHQFCKSMVKNYMDSEIYLPDQEEMAEISKSFEDKSDIPLVIGAMGRLHVPITPSLADSKNYINGRKWPSLILQAVVDNNHCFRHITCGHVGATEDSVVLGDSGLYQHFDGADMPSQNINGNTVKSFIVSDTVYPLLPWLQHGYMTPTTTEEETFNEHLNKARVAVDEAFDRLRARFRILQRKIDIDINFVPQILLTCCILHNFLEKNNTPFLEAWSEALQESNQKYPQPDGAASLNYTTTVEGESIRDVLKEHLHSKYVLFRTIEYNQVYFISEGNN

>A_christyi_ APLG2

MDSAHKRQVVENLFLTFATKQAELFKQYDANRKKRSKIFLQRLRYQLKRNGENHRKEWSSKLEAELLESIPTRKIWKLSRNDKWFRELFEQEDNETELLQYFRMGREMYDTLVAMLRQDLAPHPLLAAQSCSAEKKVGIGLYKLTTGADYTTIGNMFGVHKATVKNCVHQFCKSMVKNYMDSEIYLPDQEEMVEISKSFEDKSDIPLVIGAMGRLHVPITPSLADSKNYINARKWPSLILQAVVDNNHCFRHITCGHVGATEDSVVLGDSGLYQHFDGADMPSQSINGNTLKSFIVSETSFPLLPWLQHGYMTPTTTEEETFNEHLNKARVSVDEAFERLRARFRILQRKIDIDINFVPQILLTCCILHNFLEKNNTPFLEAWTEALQESNQKFPQPDGSASTNYTTTVEGESVRDVLKEHLHSKYVLFRTIEYNQVYFISEGTN

**APLG2b protein sequences in *Anopheles***

>A_darlingi_APLG2b

MHKVALVSPMSSLKQFQREYGALLDELIREQAELCERFESLRRRRHIIFFKRLRYKIKCKLERHREEWVEMLRKTLHENVPTRPIGRPARDDSWYRSQFEAGDDATLLATIRMDRRTYLYLVDVLRGDLTPQSQQILAPRETVSVEKRCAIGLYKLASGENFSQLGKRFGVHKLAAADSFYRFCSTIVTRLRRNVIRLPSEQEALKLAVRFEKNTCLPRVMALIGMMHFPITNAPTNSYINNDGWASMLLQAVVDHSGLIRFATSNHAGSTDAYTVLDESEVNNHFNHSELPTEFYIGTDDQAAVKPFLIGGSEYPLLPHLITRYSAPESREELLFNKHLDAALGAFDVTMQRLMARWNILHRGMDFDPVTVPRTIVCCCILHNILERRSISFDTNWITRKRYDIQPTAKPPEQLVAQPDGEVIRDWAKRRLAIRLAKSEAEAKKPPYRTIQQRVQLPK

>A_albimanus _APLG2b

MYKVALVSSMSVKQFRREYGALLDDLTREQTELCERFESLRRRRHIIFYKRLRYKIKCKLERHREEWIEMLRKTLHENVPSQTVGRPVRDDSWYRSRFLDGDDATLLATIRMDRRTYLYLVDVVRGDSTPQSQEILAPRETVTVEKRCAIGLYKMASGESFSQLGARFGVHKLTAANSFYRFCSTIVTRLRRNVIRLPAEQEALKLAVRFEKNTGLPRVMGLIGMMHFPIANAPNNSYINGDGWASMILQAVIDHSGLIRFATSNHAGSKDAYTVLEESEVNNHFNHSELPTEFYIGPDDQAAVKPFLIGGREYPLLPHLITRYPAPESREELLFNKHLDAALGAFDVTMRRLMARWNILRRRMDFDPLTVPRTIVCCCILHNILERRSIPFEMHWITRDRYDIQPTVKPPERVVAQPDAEVIRDWAKRRLVIRLSKDEAKKAPYRTAQQRIYLRK

>A_aquasalis_APLG2b

MYKVALVSPMPLKQFRREYGALLDDLTREQTELCERFESLRRRRHIIFYKRLRYKIKCQLEQHREEWVEMLRKTLHENVPTRTIGERVADDSWYRSHFLDADDATLLATIRMDRRTYLYLVDVVRGDLTLPSQDPQAPNETVSVEKRCAIGLYKMASGECFTQLGTRFGVHKLTAANSFYRFCTTIVSRLRRNVIRLPAEQEALKLAVRFEKNTGLPRVMGLIGMMHFPITKAPTNGYINSDGWASMILQAVVDHSGLIRFASSSHAGGTDGYTVLEESEVNNHFNHSELPTEFYIGDQAADDQAAVKPFLIGGREYPLLPHLITRYSAPESREELLFNKHLDAALGAFNVTMRRLMARWNVLRRGMDFDPLTVPRTIICCCILHNILERRSIPFDMQWITADRNDTQPTAKPPEQVVAHPDAEVIRDWAKRRLAIRLAKDEAKKPPYRTVQQRAN

**APM1 protein sequences in *Anopheles***

>A_gambiae_APM1

MAFKIETSNQDTTFWYEFIELYRNLPILWSDAHENKPKHLRDRALNILLRKYRETDAAAGVLEIKQLLKDLRLAYRTELKLALEHTYATGNSLSNYRSALWYFEALSFLESKELRTLKLHSGLTGDNYGIVIDTSFSTIDDSFSCINNYDDEIVLYCKSLVPALKRLPPQQLMYAKYHIDNVVKWGRIGILTYDRSHIDADGSKKSSGAVNVENCTEVLTTQNDISVPPKRLKVDQHNSDDANHATTSLAYDMCKHISMPSEECLVVVDVSENDIEQFKLASVDNEFDNLSFETSSPTYARPDGTSDSDRIIHLEISHSDLEDGADPYETADPHETADPHETANPHETADPHETTLKNGNQCF

>A_coluzzii_APM1

MAFKIETSNQDTTFWYEFIELYRNLPILWSDAHENKPKHLRDRALNILLRKYRETDAAAGVREIKQLLKDLRLAYRTELKLALEHTYATGNSLSNYRSALWYFEALSFLESKELRTLKLHSGLTGDNSGIVIDTSFSTIDDSFSCINNYDDEIVLYCKSLVPALKHLPPQQLMYAKYHIDNVVKWGRIGILTYDRPHIDADGSKKSSGAVNVENCTEVLTTQNDISVPPKRLKVDQHNSDDANHATTSLAYDMCKHISMPSEECLVVVDVSENDIEQFKLASVDNEFDNLSFETSSPTYARPDGTSDSDRIIHLEISHSDLEDGADPYETADPHETADPHETADPHETADPHETTLKNGNQCF

>A_bwambae_APM1

MAFKIETSNQDTTFWYEFIELYRNLPILWSDAHEIKPKHLRDRALNILLRKYRETDAAAGVREIKQLLKDLRLAYRTELKLALEHTYATGNSLSNYRSALWYFEALSFLESKELRTLKLHSGLTGDNSGIVIDTSFSTIDDSFSCINNYDDEIVLYCKSLVPALKHLPPQQLMYAKYHIDNVVKWGRIGILTYDRPHIDADGSKKSSGAVNVENCIEVLTTQNDISVPPKRLKVDQHNSDDANHATTSLAYDMCKHISMPSEECLVVVDVSENDIEQFKLASVDNEFDNLAFETTSPTYARPDGTSDSDRIIHLEISHSDLDDGADPHETADPHETADPHETTLKNGNQCF

>A_arabiensis_APM1

MAFKIETSNQDTTFWYEFIELYRNIPILWSDAHENKPKHLRDRALNILLRKYRERDAAAGVREIKQLLKDFRLAYRTELKLALEHTYATGNSLSNYRSALWYFEALSFLESKELRTLKLHSGLTGDNSGIVIDTSFSTIDDSFSCINNYDDEIVLYCKSLVPALKRLPPQQLMYAKYHIDNVVKWGRSGILTYDRPHIDADGSKKSSGAVNVENCTEVLTTQNDISVPPKRLKVDQHNSDDANHATTSLAYDMCKHISMPSEECLVVIDVSENDIEQLKLASVDNEFDHLSFETSSPTYARPDGISDSDRIIHLEISHSNLEDGADPHEMADPHETTLKNGN

>A_merus_APM1

MAFKIETSNQDTTFWYEFIELYRNLPILWSDAHENKPKHLRDRALNILLRKYRETDAAAGVREIKQLLKDLRLAYRTELKLALEHTYATGNSLSNYRSALWYFEALSFLESKELRTLKLHSGLTGDNSGIVIDTSFSTIDDSFSCINNYDDEIVLYCKSLVPALKRLPPQQLMYAKYHIDNVVKWGRIGILTNDRPHIDADGSKKSSGAVNVENCIEVLTTQNYIAVPPKRLKVDQHNSDDANHATTSLAYDMCKHISMPSEECLVVVDVSENDIEQFKLASVDNEFDNLSFETSSPTYARPDGTSDSDRIIHLEISHSDLEDGADPHETADPHETTLKNGNQCF

>A_quadriannulatus_APM1

MAFKIETSNQDTTFWYEFIELYRNLPILWSDAHENKPKHLRDRALNILLRKYRETDAAAGVREIKQLLKDLRLAYRTELKLALEHTYASGNSLSNYRSALWYFEALSFLESKELRTLKLHSGLTGDTSGIVIDTSFSTIDDSFSCINNYDDEIVLYCKSLVPALKRLPPQQLMYAKYHIDNVVKWGRIGILTYDRPHIDADGSKKSSGAVNVENCIEVLTTQNDISVPPKRLKVDQHNSDDANHATTSLAYDMCKHISMPSEECLVVVDVSVNDIEQFKLASVDNEFDNLSFETSSPTYARPDGTSDSDRIIHLKISHSDLEDGADPHETAGPHETTLKNGNKWF

>A_melas_APM1

MAFKIETSNQGTTFWYEFIELYRNLPILWSDAHENKPKHLRDRALNILLRKYRETDATAGVGEIKQLLKDLCLAYRTELKLALEHTYATGNSLNNNRSALWYFEALSFMKSKELRTLKLHSGLTGDNSGIVIDTSFSTIDDSFSCINNYDDEIVLYCKSLVSALKRLPPQQLMYAKYHIDNVVKWGRIGILTYDRPHIDADGSKKWSGAVNVENCTEVLTTQNDISVPPKRIKVDQHNSDDANHATTSLAYDMCKHISMPSEECLVVVDVSENDIEQFKLASVDHEFDNLSCETISPTYARPDGTSDSDRIIHLEISHSDLEDGDPHETADPHESADPHETALKNGNQCF

>A_christyi_APM1

MSLIETSTRDINYWFEFIELYRNLPILWSNADDNKPKHLRDRALNILLRKYRETNPTARVGEIRKLLKALRLEYLTELKKAVEYAHATGNSFSTYQSTLWYFEALSFLESQEIGKMKLQLDLTEDESGPSRDTSFITIDDSHSSTNNCEDDIILYCKSLAIALKRLSPHQLIYAQYHIDRVVKCGTIGILNYEWSQTNIHGSDKPTGALNTVKCTEELTLLNANSIHAVPQKYLTIGQQNCDAEDAECVTASPAYDMCKHISIPHEECLVVEDMSENDIEQYKLEPFGIDHDRLSCESNASRCDHSNGTSDRVIHLEISQQHIAGIHDATLSNGKKIDSLLVVLALNLILAKMYVVSHHIYVYMFYSFLSNLSYIF

**APM2 protein sequences in *Anopheles***

>A_gambiae_APM2

MARRNVWTPEETRELLTIIKELDLMKLFGEERNTKLYQITENEMKQRGYFDKDAFQIEHKWKNLKRSYYKTKRENYLAESCEYFEELDELMAMKPPAPSSSKGKENRPANRSDRERCWKTLTCCESASQPKRPRAVLENFDVLLTKLAKVDRENNEEFFKKQKDLVDYEFDLFTHDERQYTTKVSQMLNRNMNDFCVKAQQILLQEGVVIRIIDETPTMEDSTEELELQCSDQLAHPVVKKEIVKEFYQTWREEEPF

>A_coluzzii_APM2

MARRNVWTPEETRELLTIIKELDLMKLFGEERNTKLYQITENEMKQRGYFDKDAFQIEHKWKNLKRSYYKTKRENYLAESCEYFEELDELMAMKPPAPSSSKGKESASQPKRPRAVLENFDVLLTKLAKVDRENNEEFFKKQKDLVDYEFDLFTHDERQYTTKVSQMLNRNMNDFCVKAQQILLQEGVVIRIIDETPTMEDSTEELELQCSDQLAHPVVKKEIVKEFYQTWREEEPF

>A_arabiensis_APM2

MARRNVWTPEETRELLTIIKELDLMKLFGEERNTKLYQITENEMKQRGYFDKDAFQIEHKWKNLKRSYYKTKRENYLAESCEYFEELDELMAMKPPAPSSSKGNESASQPKRPRAVLENFDVLLTKLAKVDRENNEEFFKKQKDLVDYEFDLFTHDERQYTTKVSQMLNRNMNDFCVKAQQILLQEGVVIRIIDETPTMEDSTEELELQSSDQLAHPVVKKEIVKEFYQTWREEEPF

>A_quadriannulatus_APM2

MARRNVWTPEETRELLTIIKELDLMKLFGEERNTKLYQITENEMKQRGYFDKDAFQIEHKWKNLKRSYYKTKRENYLAESCEYFEELDELMAMKPPAPSSSKGKESASQPKRPRAVLENFDVLLTKLAKVDRENNEEFFKKQKDLVDYEFDLFTHDERQYTTKVSQMLNRNMNDFCVKAQQILLQEGVVIRIIDETPTMEDSTEELELQSSDQLAHPVVKKEIVKEFYQTWREEEPF

>A_bwambae_APM2

MARRNVWTPEETRELLTIIKELDLMKLFGEERNTKLYQITENEMKQRGYFDKDAFQIEHKWKNLKRSYYKTKRENYLAESCEYFEELDELMAMKPPAPSSSKGKESTSQPKRPRAVLENFDVLLTKLAKVDRENNEEFFKKQKDLVDYEFDLFTHDERQYTTKVSQMLNRNMNDFCVKAQQILLQEGVVIRIIDETPTMEDSTEELELQSSDQLAHPVVKKEIVKEFYQTWREEEPF

>A_melas_APM2

MARRNVWTPEETRELLAIIKELDLMKLFGEERNTKLYQITENEMKQRGYFDKDAFQIEHKWKNLKRSYYKTKRENYLAESCEYFEELDELMAMKPPAPSSSKGKESASQPKRPRAVLENVDVLLTKLAKVDRENNEEFFKKQKDLVDYEFDLFTHDERQYTTKVSQMLNRNMNDFCVKAQQILLQEGVVIRIIDETPTMEDSTEELELQSSEQLAHPVVKKEIVKEFYQTWREEEPF

>A_merus_APM2

MARRNVWTPEETRELLAIIKELDLMKLFGEERNTKLYQITENEMKQRGYFDKDAFQIEHKWKNLKRSYYKTKRENYLAESCEYFEELDELMAMKPPAPSSSKGKESASQPKRPRAVLENFDVLLTKLAKVDRENNEEFFKKQKDLVDYEFDLFTHDERQYTTKVSQMLNRNMNDFCVKAQQILLQEGVVIRIIDETPTMEDSTEELELQSSEQLAHPVVKKEIVKEFYQTWREEEPF

>A_christyi_APM2

MARRNVWTPEETRELLAIIKDLDLMKMFGEERNTKLYQITENEMKQRGFYDKDAFQIEHKWKNLKRAYYKTKRENYLIESCEFFEELDELMAMKPVAPNNPKNRESASQPKRPKAVLENFDVLLTKLAKVDRENNDEFFKKQKDLVDYEFDLFTHDERQYTTKVSQMLNRNMNDFCVKAQQILFQEGVVIRIERDDMPTMEEGQEELELQSSEQLDQPIVKKEIVKEFYQTWREEEPF

>A_stephensi_APM2

MARRNVWTTEETRELLAIIKELDLMKLFGEERNSKLYQIAENELKQRGFFDKDAFQIEHKWKNLKRTYHKTKRENYLTESCEFYEELDELMAMKPTASSSKAKDASGQPKRPRAVLENFDLLLTKLAKVDRENNEEFFKKQKDLVDYEFDLYTHDERQYTTKVSQMLNRNMNDFCIKAHQILLQEGMVEPVVQSDEALEDVQEEQELAPTEQVMMMEQQQPLVKKEIVKEFYQTWREEEPF

>A_sinensis_APM2

MAKRNVWAPDETKELLAVIKDLNLMKCFGEERNIKLYETVQNELKNRGYHGKEASQIEHKWKNLKRAYYKTKRENYLTESCEFYDELEELMSMRGSFTPKPKEGIKRTKPVLESFDILLAKITKAEKENNEEFTRKEKDLIDYQFELYSQDERQSTSKLSQLLTKNMEDFCARTQQVLAEEGLLTSNVTLLEITEQTDEGELVGSTVKKEIVKEFYQTWREEETT

>A_darlingi_APM2

MVPPRTLWSDDETFDLLDIIKLQNVDETFLLGDPASRHKHDAIYRNIAKEMWLRGYIDKHADDVQLRWEKLQRCYHKAVRVDDWTIAGPFYEELHELLSAERKRRPVDPTLREKTPKPKTVEEPTDSKSSETNEPQSGTKGMITRRKILKKIKNFRYKEDGAYYKQCRELNESGATLYGRFSKNRDYKFNGILDKNSRNIITHFRRMVGRKIATNDEAEDPNDHLNGDEDCTYEVVISES

>A_albimanus_APM2

MVPPRALWSDDETFDLLDIIKLQNVGEAFLLGDPSSGHKHEAIYKSIAKEMWLRGYIDKHADDVQLRWEKLLRCYHKAVRVDNWTIAGPFYEELHELLSEERKSRPGDNSQHRQEAPKPKPTVESNDSKSSETTESQSSTKGMATRRKILKKIKTFRYKEDGAYYRQCRELNEFGATLYGRFSKNRDYRFSGLLDKNNSNIIKQFRRMIDGKRIGESATKRAAAEDQNDRQNRDEDCTYEVVVSEC

**APM2b protein sequences in *Anopheles***

>A_darlingi_AMP2b

MVPPRTLWSDDETFDLLDIIKLQNVDETFLLGDPASRHKHDAIYRNIAKEMWLRGYIDKHADDVQLRWEKLQRCYHKAVRVDDWTIAGPFYEELHELLSAERKRRPVDPTLREKTPKPKTVEEPTDSKSSETNEPQSGTKGMITRRKILKKIKNFRYKEDGAYYKQCRELNESGATLYGRFSKNRDYKFNGILDKNSRNIITHFRRMVGRKIATNDEAEDPNDHLNGDEDCTYEVVISES

>A_albimanus_AMP2b

MVPPRALWSDDETFDLLDIIKLQNVGEAFLLGDPSSGHKHEAIYKSIAKEMWLRGYIDKHADDVQLRWEKLLRCYHKAVRVDNWTIAGPFYEELHELLSEERKSRPGDNSQHRQEAPKPKPTVESNTDSKSSETTESQSSTKGMATRRKILKKIKTFRYKEDGAYYRQCRELNEFGATLYGRFSKNRDYRFSGLLDKNNSNIIKQFRRMIDGKRIGESATKRAAAEDQNDRQNRDEDCTYEVVVSE

> A_aquasalis_AMP2b

MEPPRALWSDDETFDLLDIIKLQNVSEAFLLGDPSLRHTHEAIYKSIAKEMWLRGYIDKHADDVQLRWEKLLRCYHKAVRVDDWTIAGPFYEELHELLSEERKSCPGADPTPKPKAIEEPTDSKSSEMNESPQGSTKGMITRRKILKRIQSHRYKEDGAYYRKCRELNEFGATLYGQFSKNRDHKFNGLLDKNSSNIIAQFRRMVDGKSVAKRATSKAAAAEDANDHRNGDEDCTYEVVVSES

**LPLG1 protein sequences in Lepidoptera**

>Zerene_cesonia_LPLG1

MAFDKEFIMELFLEDNVSSVNRVIIAAANVIKEDHENITRINDIVRNECAIQVKQHDNNTLYRDTQITYEDNKFYENIVVNYTDEDYAAKFMMKKSTVQALINHLKDYIKPGACIIPLDKKVHIFLWLLTSDSSFNDVAKLFSLHKSSVSYIFHEIATLLSEQRYHFINWPSLEEQHVTRIKVNSRYGFPNCVGFIDACRLKVGSKLNRKNKPEIILLQAVCDETLMFFDIHIGEIGNTHKSRVYKDSQLAHELKNFIDFDNHILGNSEYKLKKNLITPFTSDEVLTSEEMKFNEIHWKTCTYIGHAFELLKERFKKLNHIDINKAESIKTLICAACILHNFVLLHEGSSYMKEEAVINDDGVTIDPSIVKTALEKRQFLCNYINYIDDS

>Pieris_rapae_LPLG1

MDFDKELLMELFLDDNVSNINRLIVAAANVINENNENISSINEIIMKEDANQIIRTETKEFVRRSLSYSSKNFYEDIVVKYTDEDYAEKFKMKKTTIQALINHLREYIKPCSSVIPLDKKVHIFLWLLTSDSSYSDVAELFGMHKSSVSYIFHELATLLSEQKYHFINWPSLEEQHVTRIKVNSRFGFPNCVGFIDACRLRVRSKSNQKGKPEIILLQAVCDETLMFFDIHIGQLGNTHKNRVYRESQLAHEMKNFIDFDNHILGNSEYKLKKNLITPFTSDIPLTSEETKFNEIHWKASTYIGHAFEILKDRFMKLNSIDINKTDAIHTLICAACVLHNFVLLHEGSNYLKEEAVINNDGVTIDPNLVKTAAEKRKFLCSYINYMDGA

>Vanessa_tameamea_LPLG1

MFIKENNKYLTKKQNMLSNFATEFVTELFLDDNVSTMNRLIVATANVVNENFENVTCVNDILLKEYNQISPLASGRFVKSLREGRHFYEKIVKKFTDDEYIENFKMRKATVQALIMFLKEYVKPSSSIVPFDKKVHVFLWLLTSNYSFKEAGEKFGLHKSSISYIFHEIANLLSEQRYQFINWPSLEEQHVTRVKVNSRYGFPNCVGFIDACRLKVGSKRKMKDKPEFILLQAVCDETLMFTDIHVGEIGNTRKGKVFKESQLAHELKNFLDLDNHILGDSDYKLRRNLLTPFTSDELLTSEEMKFNEIHWKAHSYINQAFEILRGKFMKLNHIDISKPESITTLIYAACVLHNFILLHEGSPVKEEAVSNDEGITIDTNVVTTALEKRQFLCNYINYIQNLQY

>Danaus_plexippus_LPLG1

MSNFNTEFIMELFLDDNVSTLNRLIVATANTVNENLENISYIKRTLLKQQGIIVQPSDQFRSVKDVTIPYENKNFFELIVKNFTNEEYIIKFKMQKATVQALITFLKDHFKPGGSIVPLDKKVHVFLWALVNDCSFKEVGQIFGLHKSSVSYIFHEVVMLLAEQRYQFINWPSLEEQHITRVKVNSKYGFPNCVGFLDACRLKVGSKRKKRQVSNFILLQAVCDETLMFIDIHIGEIGNTVKGKVFRETQLSQELKNFIDFDNHILGDCEYKLRKNLLIPFSREELVSNEEIKFNEIHWKAHSYIGSAFGLLKERFQKLNHIDINRPDAVQALICAACVLHNFVLLHEGTPVKEEAVINDEGVSIDTNVVKSAVEKRQFICNYINYIHNLS

>Spodoptera_litura_LPLG1

MNNFQKEFIMDMFLDSDHACTMNRLIVATANVISENNDNLNMFSTAVEKEFDLTSLAEENQSPGVVPVTRRLKGFYENSVRNFTDADYSAWFKIKKSTVQALINFLKKYVKPGNISLEKKVHVFLWLMVSDCSYNEIGKLFGLHKSSVSYIFNEIGTILTEHRYSFISWPSVEEQHITRIKVNSRFKFPNCVGFIDACRFKVGSKRNKKDKPEIILLQAVCDESLMFLDIHIGEVGKTRKNRVFKESTLSHELKNFIEFENHILGDSEYKLKKNLITPFTSEELLTSEEMKFNEIHWKTRSYIGHAFELMKERFRKLNHIDIVKVESVHLLISVACILHNFILIHEGSSEVKEEAVACDDGVSINTSIVQTALEKRQFLCNYINYIDTEEVG

>Spodoptera_frugiperda_LPLG1

MNNFQKEFIMDMFLDSDHACTMNRLIVATANVISENNDNLNMFSTAVEKEFDLTSLTEENQSPGAVPVTRRMKGFYENSVRNFTDADYTAWFKIKKSTVQALINFLKKYVNPGNISLEKKVHVFLWLMVSDCSYNEIGKLFGLHKSSVSYIFNEIATILTEHRYSFISWPSVEEQHITRIKVNSRFKFPNCVGFIDACRFKVGSKRNKKDKPEIILLQAVCDESLMFLDIHIGEVGKTRKNRVFKESTLSHELKNFVEFENHILGDSEYKLKKNLITPFTSEELLTSEEMKFNEIHWKTRSYIGHAFELMKERFRKLNHIDIVKVESVHLLISVACILHNFILIHEGSSEVKEEAVACDDGVSINTSIVQTALEKRQFLCNYINYIDSEEVG

>Spodoptera_exigua_LPLG1

MNNFQKEFIMDMFLDSDHACTMNRLIVATANVISENNDNLNMFGAAVEKEFDLTSFTEETKSPGVVPVTRRLKGFYENSVRNFTDADYSAWFKTKKHTVQALINFLKKYVNPGNISLEKKVHVFLWLMVSDCSYNEIGKLFGHKSSVSYIFNEIATILNEHRYSFISWPSVEEQHITRIKVNSRFKFPNCVGFIDACRFKVGSKRNKKDKPEIILLQAVCDESLMFLDIHIGEIGKTRKNRVFKESTLSHELKNFIEFENHILGDSEYKLKKNLITPFTSEELLTSEEMKFNEIHWKTRSYIGHAFELMKERFRKLNHIDIVKVESVQLLISVACILHNFILIHEGSSEVKEETVACDDGVSINTSIVQTALEKRQFLCNYINYIDTEEVG

>Helicoverpa_armigera_LPLG1

MNNFQKEFIMGMFLDDDNASTMERLVVAVANIVSENNENLNKFSTAFEKEFDYTSVRVENPGVVPVTRRMKGFYEDSVRNFTDDDYAVRFKMKRSTVQILINFLKNYVKPGNISLDKKVHVFLWLMVSDSSYNEIGKLFGMHKSSVSNIFHEIATLLTEHRYRFISWPSVEEQHITRIKVNSRFKFPNCVGFIDACRFKVGSKRNKKDKPEIILLQAVCDESLIFLDIHIGELGKTRKHRVFKESALAHELKNFVEFENHILGDSEYRLRKNLITPFTSEELLTSEEMKFNEIHWKTRSYIGHAFELMKERFRKLNHIDIVKLESVHLLITVACILHNFILMHEGCSEVKEEAVACDDGVTINTSIVQTALEKRQFLCNYINYIDKEDI

>Heliothis_virescens_LPLG1

MNNFQKEFIMGMFLDDDNASTMERLVVAVANIVSENNENMNKFSTAFEKEYDYTSVRVENPGIVPVTRRMKGFYEETVRNFTDEDYAVRFKMKRSTVQTLINFLKNYVKPGNIPLDKKVHVFLWLMVSDCSYNEIGKLFGMHKSSVSNIFHEIATHLTEHRYRFISWPSVEEQHITRIKVNSRFKFPNCVGFIDACRFKVGSKRNKKDKPETILLQAVCDESLIFLDIHIGDIGKTRKHRVFKESALAHELKNFVEFENHILGDSEYRLRKNLITPFTSEELLTSEEMKFNEIHWKTRSYIGHAFELMKERFRKLNHIDIVKLESVHLLITVACILHNFILMHEGCSEVKEEAVVCDDGVTINTSIVQTALEKRQFLCNYINYIDKEDI

>Maniola_hyperantus_LPLG1

MSYRDIEFIMELFLDDNVSTTNRLIVATANVISENLENISYVNDVLMKEHRPALDSVSSRRSFCLPVLREEDFYEETVKNFSEEDYYDRFKMSKTTVEALINFLKEYVKPGSSIVPLNKKVHVFLWLLTNDVSFKEIGELFGLHKSSVSYIFHEIATLVAEQRNQFINWPSLEEQHVTRIKVNSKYGFPNCIGFIDACQLKVGSRRKRRAKPEIILLQAVCDETLMFIDIHVGEIGTTRKGKVFRESFLSQELKNLVNFDNHMLGDADYKLRMNLITPFTRDEAVTSEEMRFNAVHWKAHSYIRQAFDILRDRFRKLNLIDINKPEAVNTLICAACVLHNFVLIQEGSPVKEEVVLIDEGITIDTDVVDTAAEKRQFLSNFINYFHNSKTFTNQLVD

>Trichoplusia_ni_LPLG1

MNNFQKEFMMSMFLDNDNVCTINRLVVAVANIVNENNENINTFYKSLPKEFHQTSQTGDNRCSLTPITRRIKGFYENTVRNYTDEDYIVKFKMRKSTVQALIKFLKNYVKPGIISLDKKVHVFLWLLVSDCSYNDVGNLFGMHKSSVSYIFHEIATHLKEQRYNFVTWPSVEEQHITRIKVNSRFKFPNCVGFIDACRFKVGSKRNKKDKPDIVLLQAVCDESLMFIDIHVSEVGKTKKNRVFKESPLAQELKNFVEFENHILGDSDYKLRKNLITPFTSEELLTSEEMKFNEIHWKTRSYIGHAFEIMKERFRKLNHIDIVKPESVQLIIYSACILHNFILMHEGCSEVKEEAVTCEDPVTINTSIVQNAIEKRQFLCNYINYIDKEDP

>Manduca_sexta_LPLG1

MKARNIKLLKQKAQRIYIGILLSQLTKFIMIMTNKNYKKMKNVDKEFIMDLFLDSENVCTVNRLVVSIANVINENHDNLNTLGDLMKREYNQVSQSVSDECLLGVSIRRVKCFYELVIQNFTDIEYVNKFKMKRSTVEALITFMKSYIKPGNIPLDKKVHVFLWFLLNDSSYNDIGKMFGLHKSSVSYIFNEIASLLTENRYHFISWPSSEEQHITRIKVTSRFKFPQLVGFIDACRFKVGPQRNRKEKPEMVLLQAVCDESLMFMDIYIGEKGKLRKNRVFKESPLSHELKNFVDFDNHILGDSDYRLKMNVITPFTSEELLTSEEMKFNEVHWKARSYIGRAFELLKERFRKLNHIDVVKMESVNYLISAACVLHNFILMQEGCSDVKEEIVLCDDGVSINTDIVKTAEEKRQFLCNYINYMDLEE

>Ostrinia_furnacalis_LPLG1

MDGFDKEFITQMFLDSDHVSTVNRLTVAIANVVTEHSENIIVFNEALEKDFGQVLQAVDSGIESCPDSLKVNGYYENIVRSFDNEDYLARFKMKKSTVQALINFLKENLERNTVVPLDKKVHIFLWLLTSDCSFNEVATLFGLQKATVSYIFHKMALLICEQRYNFISWPSIEEQHVTRIKVNSRYKFPNCVGFIDACHFKVGAKHRTKGKQDTVLLQAVCDESLMFIDIHIGDIGRTKKNKVFKDSSLAQEIKNFIDFENHILGDAEYKLRKNLMTPFSSEELLTSEEMKFNEVHWKARSYIGHAFELLKERFRKLNYMEVVKPEVVNTLVCAACVLHNFILLHEGCPEIKEEMIICDEGVTIDTNIVKTASEKRQFLCNYINYINLDNALTSPVNDL

>Arctia_plantaginis_LPLG1

MNNFQKEFLMNMFLDSDNDCNMNRLVVAVANIVNENCENMIRLSNVQQEAAQSSLTDFRQYFGALPLPRRVNGFYEQAILNFTEEDYIGKFKMKKTTVQALIKFLKNHVSPGNIPLEKKVHIFLYVLVSDSSCNEIAKLFGIHRSSVSYIFHEIATHLNENIYQFISWPSKEEQHVTRIKVNSRFKFPNCVGFIDACRFKVGSKRNKRDKPDIVLLQAVCDETLMFIDIYISPIGRTKKNKVFKDSQLVNELKKFVEFENHIIGDSEYRLRKNLITPFTSEELLTSEEMKFNEIHWRTRSYIVHAFELMKEKFRKLNHIDIVKPESVHLLISVACILHNFILMHEGCSEVKEEMVSYDDGVTINTSIVQTAEEKRQFLCNYINFIETEDVIS

>Bicyclus_anynana_LPLG1

MNNMDTEFVMELFLDDNVSTLNRLIVATANVVNENVENIAYINDSLMKEQNETVASVTSRHLTTIDSPLMIEHDFYEDTLKNFSQEDYYERFKMNKTTVEALINFLNVYVKPGSFIVELDKKVHVFLWLLTNDVSFKEIGELFGLHKSSVSYIFHEIATLIAEQRNQFINWPSLEEQHVTRIKVNSKYGFPNCIGFIDACRLKVGSRRKTRLKPEIILLQAVCDERLMFIDIHVGDIGITRKGKVFRESFLSEELKNLVDFDNHLLGDSDFKLRMNLITPFNCEEVVTSEEMRFNATLWKAHSYIHQAFDILRDRFRKLNLIDINKPAAVHTLICAACVLHNFVLIHEGSPVKEEVVTTDEGVTIDTDVVVTAAEKRQFLCNYINYMQNPKNF

>Leptidea_sinapis_LPLG1

MSLFDTNFVMELFLDDNVSNMNRAVVAAANVFYENYENISYIENIFRKECRSRKNVSLISKNVVFEKRNIRNKKQTYEDFTIKYSDTFYVTKFKMHKKTMQSLLNYLKDFYTNASLSIIPLSIKVHIFIYHLTTDTSLSEVSEYFGISKSSVSYIFHEIATLLSEQRFNFINWPSLEEQHITRVKVNSRCGFPNCVGFIDSSRLKVGSKQNQGNSKIVLLQAVCDENLMFFDIHIGEIGNTHKNKVFKDSQLGHELKNFIEFDNHILGSTEYKLRKNLITPFIPDEPLTNDELRFNEIHRKASTHIGQAFDLLRQRFKKLNNITLNKPEAVKKLITAACVLHNFILLHEGSTNLKEESVNIDEGLTIDRNLVQTAVEKRLFLCNYINFMEESI

>Hyposmocoma_kahamanoa_LPLG1

MDNFEKKFVMEMFLDSDSISTVNRLIVATTDVIVENIESISIFTELLNKEHFQTVRDIEICGASPSPPIQKKRQKVNIEHTVLNYSDSEYFDIFKMRRGTVQALINYLREFDKTAGTTVTLDKKVHVFLWLLTSDSSFSEIAILFGLHKSTVAYIFHEIASLLAEQRHNFISWPSVEEQHITRIKVNSRYKFPNCVGFIDACRFKVGSKRNKKDEPEVIMLQAVCDESLAFIDVHVSDIGVTKKSKVFRESWLCQELKNLIDFDNHLLGDSEYKLKKNLITPFNSEEVLTPEEMKFNDRHWKARSYIGLAFELLKDKFRKLSHIDINKPDAVQTLLYAACVLHNFIILQEGCPNLKEEPITCDDGVTIDSDIVKTADEKRQFLCNYVNYIDSA

>Galleria_mellonella_LPLG1

MNRFDKEFITELFMDSDSVFTVNRLVLAVANVINDNNDNITNFNEELNEEFQVLKTISTTDINATSVAKGVNSYYENTIKKFDDYEYMNKFKMKKATVQALVHFLAEHVICDGTVIVPLEKKVHIFLWLMTNDTSYQDTGLLFGLHKSSVSYIFQEIASALSDQRYNFINWPTIEEQHITRLKVYSRFKFPNCVGFLDACRFKVGSRRKKNNVLDTVLMQAVCDESLMFIDIHIGSVGKTKKSKVFRESQLSQELKNFIHFDNHILGDSEYKLKKNLITPFSSEELLTSEEMKFNEVHWKARSYIGHAFELLKERFRRLNHIDVCKPEFVATLIYSACVLHNFLLLHEGYPEMKEEAVVCDEGVTIDSNIIKTAIEKRQFLCNYINYMNMDNI

>Bombyx_mori_LPLG1

MYFRNMDSVEKNHKIKQEFVMELFLDTESVSIVDRLAAATANVINENHQNLTKFNDLMKTEYYQLSQMMVQDCHSGVPVAHRIKGFYEETIRKFGNNEYIERFKMKRSTVQALIAFLKPYLKPGNVPLDKKVHVFLWFMVNDSSYSDIGRLFGLHKSSVSYIFHEIALLLTEEKERFISWPSVEKQQLISNNVNSRFRFPNCVGFIDACRLKVGSQRNKREKPDIVLLQAVCDDYLTFVDIHVGDIGKTKKSKLFKDSSLSNELKIKVDFQKHILGDSEYKLKKYLITPFTSEEVLTSEEMKFNEIHWKTRSYIAHAFELLKERFRKLNHIDVLKPESVNILIISACVLHNFVLLHEGCSEVKEELITCDDGVTINSNVVKTAEEKRQFLCNYIEYMGIENN

>Bombyx_mandarina_LPLG1

MYFRNMDSVEKNHKIKQEFVMELFLDTESVSIVDRLAAATANVINENHQNLTKFNDLMKTEYYQLSQMMVQDCHSGVPVAHRIKGFYEETIRKFGNNEYIERFKMKRSTVQALIAFLKPYLKPGNVPLDKKVHVFLWFMVNDSSYSDIGRLFGLHKSSVSYIFHEIALLLTEEKERFISWPSVEKQQLISNNVNSRFRFPNCVGFIDACRLKVGSQRNKREKPDIVLLQAVCDDYLTFVDVHVGDIGKTKKSKLFKDSSLSNELKIKVDFQKHILGDSEYKLKKYLITPFTSEEVLTSEEMKFNEIHWKTRSYIAHAFELLKERFRKLNHIDVLKPESVNILIISACVLHNFVLLHEGCSEVKEELITCDDGVTINSNVVKTAEEKRQFLCNYIEYMGIENN

>Chilo_suppressalis_LPLG1

MDGFDKEFITELFLDSDSVSTMNRLVVAIANVVSENNDNLLTFNVEFTQHQQDIIKSIDFPIPRKIRCELEDFQLFQNYSDEDYISRFKLKKSTVEALITFIKEHSKKSNNTDIMLDKKVHIFLWLLISDASYSNVGLLFNLNKSSISDIFHEISSLLTELRYHFISWPSVEDQHLTRIKVNSRYKFPNCVGFIDTCRFNIGSKRKKNNKLDTVLLQGVCDENLMFTDIHVGNIGQTKKNKVFKDSLLAKELRNFVHFENHILGDSEYKLKKNLITPFSSEELLTSEEMKFNEVHWKARSYIGNAFELLKERFARLNHIDVPKGDAVNNLICSACVLHNFILSHEGCPEIKEEIVICDDGVSIDTDIVKTALEKRQFLCNYINYIDLDTTFTSDLDL

>Operophtera_brumata_LPLG1

MDNFQKEFVMGFFVDNDNSTVNRSILAMANLLLENVENLDALNEAINMENLSEFIDNSVMEPPRILKDYYEQAVVNFSDEDYEVTFKAKKTTVQALISFLRAFVPTGIIPLDKKVHVFLMFLMSDLSYNEIGKLFGLHKSSVSHIFQEIGLQLAENRYNYISWPSVEEQHVTRTKVNSRFKFPNCVGFIDTCRLKVGQRKTRHSTILLQAVCDESLMFIDIHIGEPGKTKKNKVYKDSTLAQALQNFIDFDNHILGDAEYRLKINLITPFTSEELLTSEEMKFNEIHWKARSYIGHAFELLKEKFKKLNHIDIFKPELINLLIYSACVLHNFILMHEGCSEVKEEAVLCDDCITLNTDLVMSAVEKRQFLCNYINYIDSDTLISR

>Amyelois_transitella_LPLG1

MNSFDTQFVTELFLDSNSNTTISRLAVTIANVIYENNENVNTFNEVLTNEFSNVLQSVGEHTEYHHSKNSYKCKNSGFYEKVVKTLSDVDYISNFRMKRSTAKALLQYLQENVEADKTNMLLDKKLHIFIFILTSDLSFNDVAALFCLQKSSVRNIFYDIAAMLSGLTYYLINWPSPEEQHITRLKVLNRFQFPNCVGFIDSFRFKAGSRRTKKASLDTVLLQAVCDESLMFIDIHVGSMGKTKKYKVFKESKLSQELKNFVDFDSHILGDSEYKLKYNLMTPFTVEELLTSEEMKYNEVHWKTRSYIGHAFEMLKERFKKLSHLDLDSPQAVGTILRAACVLHNFILLHEGAPVIKEEPTICDNGADEIILNIDTNIVKSSVEKRQFICNYINYMSVNNLLQGEAKEF

>Plutella_xylostella_LPLG1

MDTFDKEFIMEMFLDNESVSSMNRLIVATTDIIKENNDNAILAEEIWTKEDDSEDVELSESNHISRKRVRGFYENTVLQYSDKAYEFIFKMKRTTCQELIKFLKDYVKGGMIVPLEKKVHVFLWVLTSDASFQEVGKIFGLNKSTVSYIFYEIAQILTENRYHYISWPSIEEQHLTRVRVNSRVRFPNCVGFLDAVRLPVGPRPAPRVPRAARPRHVLLQAVCDDDLMFTDIYVGAVGRTKKHKVFKESPLYHELSNFIEFENHLLADASYKLRVNLITPFTSEEVLTDEEMKFNEAHWRARGYIGRAFELLQERFRKLRRLEAGGRLALVLGAAAVLHNLVLLHEGAPDVKQEPPGAHDAVAIDTDIVRTAAEKRQFLCNYINFIDDDSI

>Papilio_machaon_LPLG1

MNFDKDFVMDFFLDDTVSTLNKVVVAVANVVNENNENTSCFDELLIKEQSTQIIREQKVKKKSDVTDVKAYFENFVVKFTDEDYLEKYQMKKSTVQALINCLKEFQSGCTIIPLEKKVHIFLWILTSDYSFSETGVLFGLHKSSISYIFYEIASILTEQQYNYINWPSIKEQHMTRVKVNSRYKFPNCVGFIDACRLKVGSKKHRKNVSETILLQAVCDESLSFLDVHLGKIGDTRKSRVFRQSQLSKELKNFIDFENHILGDSNYRLRKNLITPFTSEELLTSEEMKFNEVHWKTRSYIGHAFELLRGRFKKLNHLDVNKQESNDTIILASCVLHNFVILHEGCSEIKHEPVICDDHIIIDREIVKTAIEKRQYLCNYINYLDSS

>Papilio_bianor_LPLG1

MNFDKEIVMEFFLDDTVSTLNKVVVAVANIVNENNENTSCFDELVIKEQSTQIIREQKQKVRKKPDVTGEKAYYENFVVKFTDEDYLEKYQMKKSTVQALVNCLKELQSGCTIIPLEKKVHIFLWIVTSDYSFSETGVLFGLHKSSISYIFYEIATILTEQRYNYINWPSIKEQHMTRVKINSRHKFPNCVGFIDACRLKVGSKKHMKNVSETILLQAVCDETLSFLDVHIGKIGDTRKSRVFRQSQLSKELKNFIDFENHILGDSNYRLRKNLITPFTSEELLTSEEMKFNEVHWKARCYIGHAFELLRERFKKLNHLDVNKQESNDTIILAACVLHNFVILHEGCSVIKHEPVICDDHIIIDREIVKTAIEKRQYLCNYINYLDSS

>Papilio_polytes_LPLG1

MNFDKDFIMELFLDDTVSTLNKVVVAVANVVNENNENTSCFDELVIKEQSTQMMQDHIQNVKKKSEVTDVKAYFENFVVKFTEQDYIEKYQMKKTTVQALINSLMEIQSGCTNVPLEKKVHIFLWILTSDYSFSETGVLFGLHKSSISYIFYEIATIVTEQRYNYINWPCLKEQHMTRVKINSRHKFPNCVGFIDACRLKVGSTRTRKNVPETILLQAVCDESLSFLDVHIGKIGDTRKSRVFRQSQLSKELKNFIDFENHILGDSNYRLRKNLITPFTSEELLTTEEIKFNEVHWKARSYIGHAFELLKDRFKKLNHLDVNKQESNNTIILAACVLHNFVILHEGCSQIKHEPVICDDHIIVDREIVTTAVEKRQYLCNYINYLDSN

>Papilio_xuthus_LPLG1

MNFDKDFVMDFFLDDTVSTLNKVVVAVANVVNENNENTSCFDELLIKEQNTQIIREKKQKVMKKSEVTNVKAYFENFVVKFTDEDYLEKYQMKMSTVQALINCLKEIQSGCTIIPLEKKVHIFLWILTSDYSFSETGVLFGLHKSSVSYIFYEIASMVTKQQNNYINWPSIKEQHMTRVKVNSRYKFPNCVGFIDACRLKVGSKRHGKNGPEIILLQAVCDESLSFIDVHIGKIGDTRKSRVFRQSQISKELKNFIDFENHILGDSNYRLRKNLITPFTSEELLTSEEMKFNEVHWKARSYIGHAFELLRDRFKKLNHLDVNKQESNDTIILAACVLHNFVILHEGCSEIKHEPVICDDHIIIDREIVKTAIEKRQYLSNYINYLDTS

>Papilio_dardanus_tibullus_LPLG1

MNFDKDFVMEFFLDDTVSTLNKVVVAVANVINENNENTSCFDELVVKEQSTQIIREQTQKVKKKPEVTDVKAYFENFVVKFTDVDYLEKYQMKKNTVQALINCLKEFQPGCAIIPLEKKVHIFLWILTSDYSFSETGVLFGLHKSSVSYIFYEIASILTEQRYNYINWPSIKEQHMTRVKVNSRHKFPNCVGFIDACRLTVGSKRNRKSVPETILLQAVCDESLSFLDVHIGKIGDTRKSRVFRQSQLSKELKNFIDFENHILGDSNYRLRKNLITPFTSEELLTSEEMKFNEVHWKARSYIGHAFELLRQRFKKLNHLDVNKQESNDTIILASCVLHNFVILHEGCSEIKHEPVICDDHIVLDREIVSTAIEKRQYLCNYINYLDSS-

>Papilio_memnon_LPLG1

MNFDKDFVMELFLDDTVSTLNKVVVAVANVVNENNENTSCFDELIIKEQSTQIIRDHNVKKKLEVTDIKAYFENFVVKFTELDYLEKYQMKKSTVQALIHSLMEIQSGCTNIPLEKKVHIFLWILTSDYSFSETGVLFGLHKSSISYIFYEIASIITEQRYNYINWPSIKEQHMTRVKINSRHKFPNCVGFIDACRLKVGSTRHRKNVPETILLQAVCDESLSFLDVHIGKIGDTRKSRVFRQSQLSKELKNFIDFENHILGDSNYRLRKNLITPFTSEELLTTEEIKFNEVHWKARSYIGHAFELLRDRFKKLNHLDVNKQESNETIILASCVLHNFVILHEGCSQIKHEPVICDDHITIDREIVKTAIEKRQYLCNYINYLDSN-

>Papilio_glaucus_LPLG1

MNFDKDFVMDFFLDDTVSTLNKVVVAVANVVNENNENMSCFDELLIKEQNSQIIRERKQLKKKSELSDVKDYYENIVVKFSDEDYREKYQMRKSTVKALINFLKDIKPGSTIVSIEKKVHIFLWLLTSDYSFSETGALFGLHKSTISYIFYDIASIITEQRYNFINWPSLEEQHMTRVKINSRHKFPNCVGFIDACRLKIGSKRNRKNVPETILLQAVCDESLIFFDVHLGKIGITRKSRLFRESQLAKELKNFIDFENHILGDSNYRLRKNLITPFTSEELLTSEEMKFNEMHWKARTYIGRAFELLRERFKKLNHLDVSKEESIDTIVYAACVLHNFIILHEGCSEIKHESIICDDGIIIDREIVKTAVEKRQFLCNYINFIDNC-

**LPLG2 protein sequences in Lepidoptera**

>Pararge_aegeria_LPLG2

MAHKALLALLGDTLSSKSDSSGSSSWSDLSSIRSEFSEDDDDEDRLFFPLMQYLIRLKRKRVDDYLHIIESWTDVEFRNRLGLSRKIAYSLIDDLEKSGCIANHKFGLKPLEPRLCFYIFLSFIANTEPLTPIATRFDISISSTFRVIRRIVAWILTKMDEAIKWPQDISDYRTICDSFHSKTGISNILGVIDCTHVKIEKPKNAREYCNPKGYYSIVLQATIDANLRFTNIYCGEPGSTSCARILRKSPIYHTASQDQSVLFPLNTFLVGHSGYPSLTWLVPPFRENKRLTLPQREFNTLHAATRKLSDKAFNLLKSRFKRVKLFTVYRNVAFITDTIVAACILHNYCIEESIFLELNAGA

>Maniola_hyperantus_LPLG2

MANKAIALLLADADTLSTYSGSSESSGWSDVSSIRSEFSEDDNDEDRLFFPLMHYIIRLRRKRVDDYLHIVESWTEVEFKNRLGLSRKLAYRLIDDLEKSGCIANHKFGLKPLEPRLCFYIFLSFIANTEPLTPIATKFDISISSTFRVIRRVVAWLLTKMNEAIKWPQDISDVRTICDSFHGKTGISNLLGIIDCTHVKIEKPKNAREYCNTKGYFSIILQATIDANLQFTNIYCGEPGSTSCTRVLKKSPIYHTATQNQSALFPHNSFLIGHSGYPSLSWLVPPFRENKRLTLHQREFNICHAAARKLSDKAFNLLKSRFRRIKLFTVYRNIAFITDTIVAACILHNYCLNEND

>Chilo_suppressalis_LPLG2

MDTQKILLLLTETTLISSDDSDTSWGDSLSIKQESDLDDDVEDPDDDRLFFPLMHYLIRLRRKRVDDYLHIIESWTDYEFKKRLRLSRKTAFRLIDDLEKSGAIASHNFGLKPLEPRLCFYIFLSFMGNTETLTPIASRFDISISSTFRVVRKIVAWILTKLNEAIKWPNDYDEITSICNSFYTKTGISDVLGVIDTTHIKIKKLKNGREYNNNKGYYSIILQATVDSNLRFTNIFCGEPGSNSCTQVLKKSPLYVTASDNRSMLFPHNTFLVGHSGYPTLPWLLPPFRENKRLTTYQRQLNCKHLQSRKISNRAFSLLKGRFRRIKVFNVYRNINFIIDTIVAACILHNYCHLENDYLDEQE

>Papilio_machaon_LPLG2

MSHKKTLIWLTDTIKFNDNSDSSSWSDMSFVKSESSEDDDEDDRLFFPLMHYLSRLRRKRLDDYLHTVDSWTDSEFKNRLRLSRKTAYTLIDELEKSGFIAQHKFGLKPLEPKLCFYIFLSFIANTEPLTPIATRFDISVSSAFRVIRRVVAWLLTKLNDVIKWPTNYIEIRTTCDDFRTNIGISDVLGVIDCMHVKIEKPKNAAAYHNSKGYFSIILQATFDANMKFINVYCGEPGSSTAARVLRKSPLYHTACQSRERLLPQNTFLIGHSGYTSLPWLVPPFRESNRLTVQQREFNTLHASARRVSNKAFKVLKNRFKRVKYFAVYRNIAFIIDTVVAACILHNYCVSENDHFNLTDS

>Hyposmocoma_kahamanoa_LPLG2

MTDHKVLIGLAETVALSSDDESSGWSDVFKSDADTSENDSEEYDDDKLFFPLMQYLIRLRKKRVDDYLHIVDSWTDLEFKNRLGLSKNTSYQLIDELQSSGFIASHKFGLKPLEPKLCFYIFLSFISSTETLIPIATRFDISISSTFRVIRRVVAWLLTKLNDVIKWPKDLNEMKSICDAFETKTRISNMLGVIDCMHVKIEKPKNATEYCNSKGYYSIILQATIDANLKFTNIYCGEPGSMNCARVLKKSPLYNTACQNASALFPQNTFLIGHSGYPLLTWIIPPFRENKRLTSSQKEFNAMHSNARKLRDKAFNALRVRFKRIKYFSVYRNIAFITDTIVAACIL

>Zerene_cesonia_LPLG2

MEKRTLAVFLSESCKDNNYSSESSDWSDTQRISDLSDDDDTNEDTLFFPLMQYLLKSKKKRVDDYLHCIESWTDVEFKNRLRLSRQTAYRLIDDLEKSGFIATHKFGLKPLEPKLCFFIFLTFIASIESLTPIATRFDISISSTFRVIRRVVAWLLTKLNETIKWPTEIDHIKDISNTFYDKTGISNVIGVIDCLHVKIEKPKNAREYCNGKGYFSIVLQATVDANLRFTNIYCGEPGSSNCFRVLKKSPLYNTAQKDKNLLFPYNVYLIGHSGYPSLSWLVPPYRENKRLTLTQREFNSLHVSARKISEKAFTLLKSRFKRIKIFGVYRNITFITDTIVAACILHNYCVNENEELED

>Galleria_mellonella_LPLG2

MEDSKMIIWLTETILSSSDDSDVSSWGEICSEKYESSEEDDESDKLFFPLMQYLLRLRRKRVDDYLHIIDSWSDTEFKKRLRISRKTAMRLIADLEKSGFIASHKFGLKPLEPKLCFYIFLSFIANTEPLTPMATRFDISISSTFRVIRRVVAWILTKLNDAIKWPQDYNEIKTICETFHVKTGISNMLGVIDCTHIRIEKPKNARQYCNPRGYFSVILQATVDANMRFTNIFCGEPGSSNCSRVLRKSPLYNTASQNRNALFPDNTFLVGHSGYPALPWLVPPFRENKRLTLQQREFNSLHSATRKISDKAFQLLKRRFQRIKLFTVYRNIAFITDTIVAACILHNYCLNENDYLEEQE

>Manduca_sexta_LPLG2

MDSRKAILWLTETVAISSDDSDTSSWSDVCTDKLESSDDENEVDDEDRLFFPLMQYLIRLRRKRVDDYLHIIDSWTDSEFKNRLRLSRKTAYRLIDELEKSGFIASHKFGLKPLEPKLCFYIFLSFIANTEPLTPIATRFDISISSTFRVIRRVVAWILTKLNEAVKWPQDFNDIQCICDMFHSKTGIPNMLGVIDCTHVRIEKPKNAREYCNPKGYFSIILQATVDANLRFTNIFCGEPGSSNCSRVLKKSPLYNTAMQNRNALFPHNTFLVGHSGYPSLPWLVPPFRENKRLTPQQREYNSLHASTRKMSDKAFTMLKGRFRRIKLFTVYRNIAFITDTIVAACILHNYCLNEHDHLEEI

>Spodoptera_frugiperda_LPLG2

MDKQKLVLWLTETAVSSDESDSSEWSDVCTVKQDVSEDEDMSDADEDTLFFPLMQYLIRLRRKRVDDYLHIIDSWTDAEFKNRMRLSRKTAYRLIDELEKSGFIASHKFGLKPLEPKLCFYIFLSFIANSEPLTPIATRFDISISSTFRVIRRVVAWILTKLNDAIKWPQTFEEISYICDAFHSKTRITHMVGIIDCTHIKIEKPKNAREYCNPKGYFSVILQATIDANLRFTNVFCGEPGSSNCTRVLKKSPLYNTASQNRNTLFPHNTFLVGHSGYPSLPWLMPPFRENKRLTPQQREFNSLHLSTRKLSDKAFSLLKGRFRRIKLFTVYRNIAFITDTIVAACILHNYCVNENDHLEEHE

>Spodoptera_litura_LPLG2

MDKQKLVLWLSETAVSSDESDSSEWSDVCTVKQDVSEDEDMSDADEDTLFFPLMQYLIRLRRKRVDDYLHIIDSWTDAEFKNRMRLSRKTAYRLIDELEKSGFIASHKFGLKPLEPKLCFYIFLSFIANLEPLTPIATRFDISISSTFRVIRRVVAWILTKLNDAIKWPQTFEEISYICDAFHNKTGITHMVGIIDCTHIKIEKPKNAREYCNPKGYFSVILQATIDANLRFTNVFCGEPGSSNCTRVLKKSPLYNTASQNRNALFPHNTFLVGHSSYPSLPWLMPPFRENKRLTPQQREFNSLHLSTRKLSDKAFSLLKGRFRRIKLFTVYRNIAFITDTIVAACILHNYCVEENDLLEEHE

>Heliothis_virescens_LPLG2

MDKQKLVLWLSETAVSSDDSDSSEWSDVCTVKPDGSEDENITDEDEDTLFFPLMQYLIRLRRKRVDDYLHIIDSWTDAEFKNRMRLSRKTAYRLIDDLEKSGFIASHKFGLKPLEPKLCFYIFLSFIANTEPLTPIATRFDISISSTFRVIRRVVAWILTKLNDAIKWPQNFEEISYICDSFHTKTGITHMVGIIDCTHIKIEKPKNAREYCNPKGYFSIILQATIDANLRFTNVFCGEPGSSNCARVLKKSPLYNTASQNRNALFPHNTFLVGHSSYPSLPWLMPPFRENKRLTPQQREFNSLHMSTRKLSDKAFSLLKGRFRRVKLFTVYRNIAFITDTVVAACILHNYCLDENDHLEEHE

>Helicoverpa_armigera_LPLG2

MDKQKLVLWLSETAASSDDSDSSEWSDVCTVKPEGSEDENMTDEDEDTLFFPLMQYLIRLRRKRVDDYLHIIDSWTDAEFKNRMRLSRKTAYRLIDELEKSGFIASHKFGLKPLEPKLCFYIFLSFIANTEPLTPIATRFDISISSTFRVIRRVVAWILTKLNDAIKWPQNFEEISYICDSFHTKTGITHMVGIIDCTHIKIEKPKNAREYCNPKGYFSIILQATIDANLRFTNVFCGEPGSSNCARVLKKSPLYNTASQNRNALFPHNTFLVGHSSYPSLPWLMPPFRENKRLTPQQREFNSLHISTRKLSDKAFNLLKGRFRRVKLFTVYRNIAFITDTVVAACILHNYCLDENDHLEEHE

>Danaus_plexippus_plexippus_LPLG2

MDKIKLTYFLTEYIHSSDDSDSSSWSDLSSVKSEFYEDDEEDRLFIPLMQYLIRIKRKRVDDYLHFVESWTDAEFKNRLILSRKTAYKLIDDLEKSGFIASHKFGLKPLEPKLCFYIFLSFIADTEPLTPLANRFDISISSTFRVLRRVVAWLLTKLDDAIKWPQDFNDVETICEQYHFKTGISNILGVIDCTHIKIEKPRNAREYCNPKGYFSIVLQATIDANLRFTNIYCGEPGSSNCSRVLRKSPLYQTATQNRDTLFPHNTFLVGHSGYPSLSWLVPPFRENKRLTSQQREFNSLHASARKLSDKAFNLLKTKFRRIKLFTVYRNIPFITDTIVAACILHNYCLDESCDPSEE

>Trichoplusia_ni_LPLG2

MDKQKLILWLSETAQSTDESDSSEWSDVCTVKQDVSEEEDFSDNDDDSLFFPLMQYLVRLRRKRVDDYLHIIDSWTDSEFKNRLRLSRKTAYRLIDELEKSGFIASHKFGLKPLEPKLCIYIFLSFIANTEPLTPIATRFDISISSTFRVIRRVVAWILTKLDDAIKWPQSYEEISYLCDSFNTKTGITHMIGIIDCTHIKIEKPKNAREYCNPKGYFSIILQATIDANLRFTNVFCGEPGSSNCARVLKKSPLYNTATQNRNSLFPHNTFLVGHSGYPSLPWLMPPFRENKRLTPQQREFNALHTSTRKLSDKAFSLLKGRFRRIKLFTVYRNIAFITDTIVAACILHNYCLNENDHLEEHE

>Vanessa_tameamea_LPLG2

MANKNLILFLSETLPDSSNDSDSSSWSDICGVGSEFSEDEDEEDRLFFPLMQYLVRLRRNRVDDYLHIVDSWTDIEFKNRLRISRKTAYRLIDDLEKSGYIAQHKFGLKPLEPKLCFYIFLSFIANTEPLTPIASRFDISISSTFRVIRRVVAWVLTKLNEAIKWPQDYNDIRTICDNFQGKTGISNMLGVIDCTHVRIEKPKNAREYCNPKGYFSIILQVTIDSNLCFTNIYCGEPGSSNCTRVLKKSPLYHTATQNRNALFPHNTFLVGHSGYPSLPWLVPPFRENKRLTIEQREFNSLHAATRKLSDKAFTILKNRFRRVKLFTVYRNIAFITDTIVAACILHNYCLKENDHLEDNE

>Bicyclus_anynana_LPLG2

MANKALALLLTDTISYKSDSSDNESSSWSDLCSLTSEFSDDDDDEEDRLFFPLMQYLIRLRRERVDDYLHIIESYTEAEFKNRLGLSRKIACRLIDDLEKSGIIANHKFGLKPLEPRLCFYIFLSFIANTEPLTPIATRFDISISSTFRVIRRVVAWILTKMDDAIKWPQDMSEIMTICDNFHNKTGISNMLGVIDCTHVKIEKPKDAPEYCNTKGYFSIILQATIDAKLRFTNIYCGEPGSLSCARVLRKSPLYHTATQNQAGLFPHNTFLVGHSGYPSLTWLVPPFRENKRLTLNQREFNSLHAATRKLSDKAFNLLKNRFRRIKLFTVYRNVAFITDTIVAACVLHNYCLSENE

>Bombyx_mandarina_LPLG2

MIKNNQIIWLAETNNFLISTDDSDTDSFSHVSNVEGNSSEIEWPDDEDDEIFFPPMQYFIRLRRRRVDDYMHIIDSWTDAEFKNRMRLSRKTAFRLIGELDKSGFIASHKFGLKPLESKLCFYIFLTFISNTEPLTPIATRFDISISSTFRVIRKVASWILTKLNDAIRWPQDFNEIQYICDNFHLKTGISNILGVIDRTHIRIDKPKNAREYCNPKGHFSIILQATIDSQLRFTNIFCGEPGSSNCTRALKKSPLYHTATHDRNSLFPHNTFLIGHSGYPSLPWLVPPFRENKRLTPQQREFNALHASARKLGDRAFILLKARFRRIKLFTVYRNIAFITDMIVAACILHNYCLNENDHLEVNHD

>Arctia_plantaginis_LPLG2

MNTNELVIFLTETVHSSSESESSGWSDVCSIKPESSEDEDERGDDDDDTLFFPLMQYLIRLRRKRVDDYLHIIDSWTDSKFKTKMRVSRKTAFRLIDELEKSGFIASHKFGLKPLEPKLCFYIFLSFIANTEPLTPIATRFDISISSTFRVIRRVVAWILTKLNDAIKWPQNLEEIQYICDAFHAKTGIANMVGVIDCTHIKIEKPKNARDYCNPKGYFSIILQTTIDANLRFTNVFCGEPGSSNSSRVLKKSPLYHTATQNKDSLFPHNTFLVGHSGYPSLPWLLPPFRENKRLTPQQREFNSLHITTRKLSDKAFSLLKGRFRRIKLFTVYRNIAFITDTIVAACVLHNYCLDENDHLEEHD

>Bombyx_mori_LPLG2

MIKNNQIIWLAETNNFLISTDDSDTDSFSHVSNVEGNSSEIEWPDDEDDEIFFPPMQYFIRLRRRRVDDYMHIIDSWTDAEFKNRMRLSRKTAFRLIGELDKSGFIASHKFGLKPLESKLCFYIFLTFISNTEPLTPIATRFDISISSTFRVIRKVASWILTKLNDAIRWPQDFNEIQYICDNFHLKTGISNILGVIDRTHIRIEKPKNAREYCNPKGHFSIILQATIDSQLRFTNIFCGEPGSSNCTRALKKSPLYHTATHDRNSLFPHNTFLIGHSGYPSLPWLVPPFRENKRLTPQQREFNALHASARKLGDRAFILLKARFRRIKLFTVYRNIAFITDMIVAACILHNYCLNENDHLEVNHD

**LPM2 protein sequences in Lepidoptera**

>Pararge_aegeria_LPM2

MDENIDNNVAVLAILDNQSSPYEQNLNITNYDQSHQINQEEIETSETSKQNIIWTKNATLMLLNLYETKLNVLDNPKKKSKMWISMAEELKSFHIEVTPDQVRWKINALTKKYKDCIDNGQGSVVFKYFNEMHQILGRYNEDSGAYRLASGVIQGPEVGKNLIAPCRKISFKNTTPFRKLRAERRAKVEIDKQWIDYIKRHEEQKQIRDERYERSLRLREEELQLKKKELEIKQSIALRKLELREKRQEEILKIEREKCALLRKLLNDH

> Maniola_hyperantus_LPM2

MDENIDNNVAVLAILDNQSSPYKQNINITNYNHLHQINQEEIETSETCKQNIIWTKNATLMLLNLYETKLNLLDNPKKKSKMWISMAEELKPFHIEVTPDQVRWKINALTKKYKDCIDSGQGSAAFKYFNEMHQILGRFNEDSGTYRLASGVIQSQEIVKDHTINGKIAFKSSTPFRKLRAERRAKVELDKLWIEYIKRQEEQKQIRDERYERSLRLREEELQLKKKELEIKQSIALRKLQLKEKRQEEILKIEKEKCALLRQLLNDR

>Chilo_suppressalis_LPM2

MNENINSNVSVLSVLDNQCSTSSVFKPDINMESYRNPNPPHNISHPHSQEQVVAHQEADNPDSNKQSILWTKNSTVTLLSFYEIKMGMCDNPKKKSKMWVSIAEELKGVGIEVTADQVRWKINALTKKYKDCIDNGQEELGFKYFNEMHQILGQNNETGGSYRLSSGMQTQDSSEKEQKKRLGMTQNNTPFRRLRAERRAKIELDKLWFDYIQKQDALKHLRDERYERSLRLREEELQLKKKELEIKQSLALKKLQLKERKHEEIIKIEREKCLLLRKLLENQ

>Papilio_machaon_LPM2

MEENIDNNVTVLAMLDNQSSTYKQNVLPDYNHSNGMKSEETETNETGKHNIIWTRNATLLLLNLYESKIHMIDNPKRKSKMWMSMAEELKALNIEVTPDQVRWKINALTKKYKDCIENGQGNLTFKYFNEMHQILGQYNDDNETYRLASGVTQCDRRQSNKNAILSCSPYHKIRTERKAKVELDKQWLDYLKRQEEQKELRDQRYERSLRLRQEELELRKKELEIKESLALKKLQLKEKMQEEILKIEREKCALLRTFLNPQ

>Hyposmocoma_kahamanoa_LPM2

MNPNMNDNNVTVFAILDNHYGGTSSEYKTNLNVNYSIQNIESNISNQDEINGNESNKQNYALSADKIWTKNATLMLLSLYETKIHMLENPKKKSKMWTAIAEELKSLNIEVTSDQVRWKINALTKRYKDCVDNGQNTSDFKYFNEMHQILGRYNDDTGTYRLASGVQRDTEVVKTKTLKAPFRKLRAERRAKVELDKQWMEYLKKQDEQKQIRDERLERSLILREEKLQLRKKELELKQNLALKKLQLKQRKHEELLRIEREKCELLMKLLDKNNKLTRILE

>Zerene_cesonia_LPM2

MDENIDNNVAVLAILDNQCDGTLESPFKQSVNIANYSHAHTISQEGTDSHETNKQNVIWTKNATLMLLNLYETKLSTLDNPKKKSKMWSTMAEELKSFNIEVTPDQVRWKINALTKKYKDCLDNGQVSGFKYYNEMHQILGKYNEDNDTYRISSGIQDQINKDNLCRKLPYKNSTSLRKLKVERRAKIELDKQWLLYLRRQEEQKHIRDERYERALRLREEEIALRKKELDIKQTVALKKLQLKEKHQEEMLKIEREKCFLLRKLLGE

>Galleria_mellonella_LPM2

MDNIDNTVSVLAMLDNQSTTYKPNMEITPFNQNHSMNQEETELSESSKQSIIWTKNATCMLLNLYETKMHMLDNPKKKSKMWTSIAEELKAINIEVTPDQVRWKINALTKKYKDCIDNGQGSTTFKYFNEMHQILGRYNNEGNAYRLASGVMQSDETVDKERLKRNLSSKSTVPFRRLRAERRAKLELDKQWIEYLRRQEEHRLERDERCERSLRLREEELQLKKKELEIKKSIALKKLQLKEKKQEEILKIEREKCALLRKLLEDQ

>Manduca_sexta_LPM2

MDENIDSNVAVLAILDNQSTAYKPTMGMATYNHVHTVNQEEIDVNESNKQNIIWTKNATLMLLSLYETKIHMLDNPKKKSKMWLSIAEELKSLNIEVTPDQVRWKINALTKKYKDCIDNGQGAMSFKYFNEMHQILGRYNDNGGNYRLASGVIQNQEDLDRDKSKRNIPYKTSTPFRKLRAERRAKVELDKQWIDYIRRQEEQKQIRDERYERSLRLKEEELQLRKKELEIKQSLALKKLQLKEKKQEEMLKIEREKCVLLRKLIADQELIRQ

>Spodoptera_frugiperda_LPM2

MDENIDSNVAVLAILDNQSAAYKPTMAMATYNHSHTITQEETDVNETNKQNVIWTKNATLMLLSLYETKMHMLDNPKKKSKMWLSIAEELKSLNVEVTADQVRWKINALTKKYKDCIDNGQGAMSFKYFNEMHQILGRYSDNTGTYRLASGVIQGSDDLDKDKNKRNMSLKGSTPFRRLRAERRAKIELDKQWLDYLRKQEDQKQLRDERYERNLRLRQEELQLRKKELEIKQSLALKKLQLKEQKQEEMLKIEREKCALLRKLVADQDLMRQ

>Spodoptera_litura_LPM2

MDENIDSNVAVLAILDNQSAAYKPNMTMTTYTHSHTITQEETDVNETNKQNVIWTKNATLMLLSLYETKMHMLDNPKKKSKMWLSIAEELKSLNVEVTADQVRWKINALTKKYKDCIDNGQGAMSFKYFNEMHQILGRYSDNTGTYRLASGVVQGSDDLDKDKNKRNMSLKSSTPFRRLRAERRAKIELDKQWLDYLRKQEDQKQLRDERYERNLRLRQEELQLRKKELEIKQSLALKKLQLKEQKQEEMLKIEREKCALLRKLVADQDLMRQ

>Heliothis_virescens_LPM2

MDENIDSNVAVLAILDNQSTPYKPNMTITNYNNHSHTITQEETDVNETNKQNVIWTKNATLMLLSLYETKMHMLDNPKKKSKMWLSIAEELKSLNVEVTPDQVRWKINALTKKYKDCIDNGQGAMSFKYFNEMHQILGRYSDNTGTYRLASGVMQGPDDLDKAKRNISLKGSAPFRRLRAERRAKIELDKQWLDYLRKQEEQKHLRDERYERNLRLRQEELQLRKKELEIKQSLALKKLQLKEQKQEEMLKIEREKCALLRKLIENQDLMRQ

>Helicoverpa_armigera_LPM2

MDENIDSNVAVLAILDNQSTPYKPNMTITNYNHSHTITQEETDVNETNKQNVIWTKNATLMLLSLYETKMHMLDNPKKKSKMWLSIAEELKSLNVEVTPDQVRWKINALTKKYKDCIDNGQGAMSFKYFNEMHQILGRYSDNTGTYRLASGVMQGPDDLDKAKRNISLKGSAPFRRLRAERRAKIELDKQWLDYLRKQEDQKQLRDERYERNLRLRQEELQLRKKELEIKQSLALKKLQLKEQKQEEMLKIEREKCALLRKLIEDQDLMRQ

>Danaus_plexippus_LPM2

MDEGIDSNVAVLAILDNQSSPYKPNLTIATYNHQLNQEEVETNETSKQNIIWTKDSTLMLLNLYETKLNMLDNPKKKSKMWISMSEELKAFHIEVTPDQVRWKINALTKKYKDCLDNGQGAVAFKYFNEMHQILGRYNEDGAYRLASGIMQADKEHNRINRKLSFKNTSPYRKLKTEHRTKVELDKQWIEYLQRQEEQRQIRDERYERSLRLREEELQLKKKELENKQSIALKKLQLKEKKHEEILKIEQEKCALLRKLLENQ

>Trichoplusia_ni_LPM2

MDENIDSNVAVLAILDNQSNQYKPNMNMNNYNFSHTITVTHEETDVNESNKQNVIWTKHATNLLLSLYQTKIHMLDNPKKKSKMWLSIAEELKSLSVEVTPDQVRWKINALTKKYKDCIDNGQGAMSFKYFNEMHQILGRYSDNSETYRLASGVMPGGSEDLDKDKSKRNNLTLKGSTPFRRLRTERRAKIELDKQWLEYLRKQEDQKQLRDERYERNLRLRQEELQLRKKELEIKQSLALKKLQLKEKKQEEMLKIEREKCALLRKLLADQDLMRQ

>Vanessa_tameamea_LPM2

MDENIDNNVAVLAILDNQSSPYKQNISLTSYNLNGHQVNQEEIETNETSKQNIIWTKNATLMLLNLYETKLNMLDNPKKKSKMWTSMAEELKSFHIEVTPDQVRWKINALTKKYKDCIDNGQGSMAFKYFNEMHQILGRYSEDNGTYRLASGVMQGQESDKDHRVINRKMSFKNSTPFRKLRAERRAKVELDKQWIEYIRRQEEQKLIRDERYEKSLRLKEEELQLKKKELEIKQSIALRKLQLKEKKQEEYLKIEREKCALLRKLLADQ

>Bicyclus_anynana_LPM2

MDENIDDNVTVLAILDNEASPYKQNISITNYDQSHQINQEEIETSETSKQNIIWTKNATLMLLNLYATKLNMLDNPKKKSKMWISMAVELKSFNIEVTPDQVRWKINALTKKYKDCIDNGQGSTAFKYFNEMHHFLGRYNEDSGAYRLASGVIHGQDTDKDHNAPNGKISYKNSTPFRKIRAERKSKVELDKQWIEYIRKQEEQKKIRDERYERCLKLREEELQLKKKELEMKQSIALKKLQLREKMQEDILKIERQKCALLKKFLNDRNNSDDIKSCYMDE

>Bombyx_mandarina_LPM2

MDENIHNNVAVLAILDSQPNLYKPNMPVPTYNHNQNISQQEESDVNEMNKQNIIWTKNSTIMLLNLYETKIHMLDNPKKKSKMWISIAEELKSLNVEVTADQVRWKINALTKKYKDCIDNGQGAVSFKYFNEMHQILGRYNDNGGNYRLASGVIQEDAEKSKDRKNLMKTTTPFRKLRAERRAKVELDKQWIEYIKRQEEQKQIRDERYERSLRLKEEELQLRRKELELKQSIALRKLQLRENKQEELLKIEREKCALLRKLVAEHEILRQ

>Arctia_plantaginis_LPM2

MDENIDNNVAVLAMLDNQSTTYKPNLPITNYNHSHTMTREESEVNETNKQNVIWTKNATLMLLSLYETKMHMLDNPKKKSKMWISMAEELKALNVEVTPDQVRWKINALTKKYKDCIDSGQGAMAFKFFNEMHQILGQYTDNSGSYRLASGVMHGQDELDKDKSKKNILLKSSTPFRKLRVERRAKVELDNQWMDYLRRQEEQKHIRDERYERNLRLREEELQLRKKELEIKQTLALKKLQLKEKKQEEMLKIEQEKCALLRKLLGIQDVLSQ

>Bombyx_mori_LPM2

MDENIHNNVAVLAILDSQPNLYKPNMPVPTYNHNQNISQQEESDVNEMNKQNIIWTKNSTIMLLNLYETKIHMLDNPKKKSKMWISIAEELKSLNVEVTADQVRWKINALTKKYKDCIDNGQGAVSFKYFNEMHQILGRYNDNGGNYRLASGVIQEDAEKSKDRKNLMKTTTPFRKLRAERRAKVELDKQWIEYIKRQEEQKQIRDERYERSLRLKEEELQLRRKELELKQSIALRKLQLRENKQEELLKIEREKCALLRKLVAEHEILRQ

**BPLG1 protein sequences in Cockroaches**

>Periplaneta_americana_BPLG1

MAVVSENVKEALLNFALTELSKSATNHVYTFVKSEMGSSRHHFLMSLLAAKDNAENSQEGGERAQKPSVSYDVLLTCSDSEFKTYFRMTRKTMQSLTRIIGKRLTASPDTVHPLVPVEKKVLLTAWLMGNNQSYMAAAKLFEISKGTVYRVFHQICSELTILAGKYVQWPDPDECDEISTSFENQYGFPGVIGVIGACHVEIREPESREEAVRFKNEQTGQYTVILQAVCDHKLKFRDVCAGFPGQTSKSRVLVGSPLYTRLSSRTDPLIEPHKHILGSSDYPQLSTLLTPYSESASGRPLTKQELKFNILHRSARSVVEQAFEILKKRFQRLRFIDVSRTELASKVVVAACVLHNFALMHGDEFDVGDKTP

> Coptotermes_formosanus_BPLG1

MEYRMDENFIAEFRDVWFRACHRSNMGAGVEDVKKALLNFAVTELSKPVTNHVYNFMKYEVGATRHHILASFLAAENSTENNIRENAARRQNPSLNYESLMTCRDADFKMHFRMNRSTIQSLTRIVGKRLTVSPEAVHPLVPVEKKVLLTVWLMGNSQSYMAAAKLFEVSKGTVYRIFHHICSELTILAGKYVQWPDPNECDEISVSFENKYGFPGVIGVIGACHVEIREPESQEEAARFKNEQTGQYTVILQAVCDDKLLFRDVCAGFPGQTSKLRVLIGSPLYTRLSSHTDPLIEPHKHILGSSDYPQLSTLLTPYSSSASGRPLTKQELKFNILHQTATSVVEQAFEVLKRRFQRLRFIDVSRTELASKVVVAACVLHNFALMHGDSFTDECDKSVGK

>Cryptotermes_secundus_BPLG1

MITMTTVMLQMAVGVEDVKEALLNFALTELSESPTNHVYTFIKSEMGAHRHHILTSLLAARDSAENKLEENTERQQTPGANYETLVTCRDSDFKTYFRMNRNTIQSLTRIIGKRLMASAEAVHPLIPVEKKVLLTAWLMGNNQSYMAAAKLFEVSKGTVYRIFHHICSELTILAGKYIQWPDPNECDEISASFENKHGFPGVIGVIGACHIEIREPEAQEEAARFKNEQTGQYTVILQAVCDDRLLFRDVCAGFPGQTSKLRVLIGSPLYTRLSSRTDPLIEPHKHILGSSDYPQLSTLLTPYSSNADGHPLSKQELKFNSLHQTARSVVEEAFEMLKKRFQRLRFIDVSRTELASKVVVAACVLHNFALMHGDEFTDEHYKNTDKKI

> Zootermopsis_nevadensis_BPLG1

MARSADGVKEALLNFALTELSKSSTNHVYNFIKSEMGSSRHHILTSLLVAKDSAENNLEEIAPRLHKPDPNYETLVKCRDADFKKYFRMNRSTIQSLVCIVEKRLIVSPEAVHPLVPVEKKVLLTAWLMGNNQSYMAAARLFELSKGTVYRIFHHICSELTILAGKYIQWPDPEESDEISVSFENKYGFPGVIGVIGACHVEIREPESQEEAARFKNEQTGQYTVVLQAVCDDKQAFRDVCAGFPGQTSKLRVLVGSPLYTRLSSHTDPLIEPHKHILGSSDYPQLSTLLTPYSTCAAGRPLTKQELRFNSLHQTARSVVDQAFEMLKRRFQRLRFIDVSRTELASKVVVAACVLHNFALMHEDEFTEECYKNTDVKI

>Blattella_germanica_BPLG1

MAAAPPGVKEALLNFALTELSSSATNHVYEFLKTDLGSSRHHMLSALLVAKDFGDSGSKEEPTEQIESPKNNYESLITCTDTDFKNIFRMSRKTIQSLIRVMNKRLAVYPDAVHPMIPVEKKVLLTAWLMGNNQSYMAAAKLFEISKGTVYRIFQQICSELTILAGRYIQWPHPEECDEISKVFEIKYGFPGVIGVIDACHIEIREPECKEESDRFLNEQTGQHTVILQVVCDHKLLFRDVCAGFPGETTKSRVLVGSPLYGRLSSRTNPLIEPDKHILGNSDYPQLSTLLTPYTATASGRALTKQEIKFNGLHRTARSVVGQAFQLLKARFLRLRFIDVSRPELASRVVAAACVLHNFALLHGDEFGEEATKGKLRADFTKRKY

**BPLG2 protein sequences in Cockroaches**

>Cryptotermes_secundus_BPLG2

MWQIILVITHWLLEYWNRRRKGYEKENWRLLRRRRLLMMRLLQQRRRRNVVKSCWAYPTSARFWEEIVPTYNDINFLKHFGTGRETFQCLVDELYLDLVREDTIMRPAIPVDKRIAVALTLLRTSSDFLSVAKLFGIGESTVHLILKEFSSAVCKTLYGKIVTFPSTVMEREEIVSAFNNRWHFPGCLGVLGTSHIPILSPSQSENNYFNKEGYHSIVVLGLVDHHHTFRYADVGQPGGSSDAETFFKSELYRYLSAKLIPEEFHLIAGSSFPLLHSLMTPFPFNKSKDSMQKRFDYHLHKAFTPLKVAFAHLRGRWKILLKRCDLQLDNMIDIAKTCLVLHNLCESNGDNYFDCWDQNVNDESTFSQSMHKENLTTASQGGVTKRNELAIFLY

>Coptotermes_formosanus_BPLG2

MWQFIFCITHWLFHYWKKRRKRYEQENWRLLRRRRILIMRLLRQRRRRYVAKSCWAYPTTGRFWEETVPTFSDISFLKHFGTGRETFQCLVDELYLDLVRENTIMRRAIPVDKRIAVALTVLRSSGDFTSVAKLFGIGRSTGHLILKEFSSAVCKTLYDKIVTFPETVTEREEITNAFNNMWHFPGCLGALGASHIPILSPSQGGDDYWNEEGYHSIVLLGVVDHQYTFRYADVGQPGGTSDSDIFFKSELYKHLCTKLIPEEFHLIAQSSFPLLHCLMTPFSFNKSKNSMQTKFDYHLHKALTPAEVAFSHLRGRWKVLLKRCDLQLDNMVDIVKTCLVLHNLCEINGDHYFDSWNESVNEETHFPQPLHQQNLTNPSQAGIKKRNELAVILP

>Zootermopsis_nevadensis_BPLG2

MWQIISVITHWLFEYWNTRRKGYEEEHGRLLRRRRLLMLRLLRQRRRRNVVKSCWAYPTTGRFWEEVVPTYSDISFLKHFGIGRETFQCLVDELYVDLVRENTNMRRSIPVDKRIAVALTVLKNSDDFLSVAKHFGIGKSTVHLILKEFSSAVCKTLYDKIVTFPATVTEREEIVNAFSNTWHFPGCMGALGTSHIPILSPSLSSSSYLNKEGYHSIVLLVVVDHQYTFRYADVGQPGGMNDAEIFLKSELYNFLCREVLPEEFHLIAGSSFPLRKWLMTPFPSNESKDSMQKRYDYHLQKATTPVEIAFGHLRGRWKILFKRCDLQLDNMINIVKTCLVLHNLCESNGDHYFDSWDENVCEERKTFVQPLHVENLTDRSQEGFSKRNELAIILY

>Reticulitermes_speratus_BPLG2

MWQIIFSITHWLFCYWKRRRKGYEQENWRLLRRRRVLMMRLLRQQRRKYVVKSCWAYPTTGRFWEETVPTFSDISFLKHFGTGRETFQCLVDELYLDLVRANTIMRRAIPVDKRIAVALTVLRSSGDFVSAAKLFGIGRSTVHLILKEFSSAVCKTLYNKIVTFPETVTEREEIMNAFNNAWHFPGCLGALGASHIPILSPSQVADDYLNEKGYHSIVLLGVVDHQYTFRYADVGQPGGTSDSEIFFKSELHKHLCTKLIPEEFHLIAHSSFPLLHCLMTPFPFNKPKNSMQKRFDYHLRKALIPAGVAFSHLRGRWKILLKRCDLQLDNMVDIVKTCLVLHNLCEINGDHYFDSWNESVNEDTQFPQPLHQQNLTNPSQEGIKKRNELVVIVS
